# Supplementary material for: COVID-19 in Pregnancy in Scotland (COPS): protocol for an observational study using linked Scottish national data
Source: BMJ Open. 2020 Nov 26;10(11):e042813. doi: 10.1136/bmjopen-2020-042813 (PMC7691999; doi:10.1136/bmjopen-2020-042813)
Supplement: Supplementary data [file bmjopen-2020-042813supp001.pdf]

Supplementary Material: Additional information on identifying pregnant women, and associated start and end of pregnancy dates, from national data sources

National data sources identifying end of pregnancy events

There are three possible outcomes for any pregnancy

Pregnancy outcome	Comments
Spontaneous loss	‘Miscarriage’ at <24w (here taken to include ectopic pregnancies, although mechanisms underlying spontaneous miscarriage and ectopic pregnancy differ)<br>(Sometime ‘late fetal loss’ at 20-23w as a subset of miscarriages)<br>‘Stillbirth’ at ≥24w
Termination of pregnancy	Legal at <24w under Grounds C and D of the Abortion Act 1967<br>Legal at any gestation under Grounds A, B, E, F, G
Live birth	No lower gestational limit although in practice around 22w would be considered the lower limit at which live born babies may survive

Various national records may be returned following these end of pregnancy events, as summarised below

National record	Description	Pregnancy outcomes identified	Coding to identify relevant records
Identifying spontaneous pregnancy losses			
SMR01	Record of day case or inpatient admission to any general unit (excluding neonatal, maternity, and mental health care), including admissions under gynaecology specialty	Will identify early (first trimester) spontaneous losses managed in hospital in most Board areas	ICD10:<br>O00 (ectopic pregnancy)<br>O01 (hydatidiform mole)<br>O02 (missed miscarriage)<br>O03, O05, O06 (spontaneous miscarriage), all .5-.9
OR<br>SMR02	Record of day case or inpatient admission to a maternity unit, including admissions under obstetrics or midwifery specialties	Will identify early (first trimester) spontaneous losses managed in hospital in some Board areas<br>Will identify later (second and third trimester) spontaneous losses managed in hospital in all areas	Miscarriages<br>Condition on discharge=2 (aborted)<br>Type of abortion=1, 2, 3, 6, 8, 9 (spontaneous)<br><br>Stillbirths<br>Condition on discharge=3 (delivered)<br>Outcome of pregnancy=2 (stillbirth)
AND/OR<br>NRS stillbirths	Record of statutory registration of a stillbirth (baby born at ≥24w showing no signs of life)	Will identify spontaneous stillbirths	ICD10:<br>P96.4 not recorded
Identifying terminations of pregnancy			
AAS	Record of statutory notification of a termination of pregnancy	Should identify all terminations of pregnancy but known under-notification of later ToPs done for fetal anomaly from some maternity units	

AND/OR<br>SMR02	As above	Will identify later ToPs done for fetal anomaly in maternity units	Condition on discharge=2 (aborted)<br>Type of abortion=4 (ToP)
AND/OR<br>NRS stillbirths	As above	Will identify the small number of stillbirths following a termination of pregnancy	ICD10:<br>P96.4 recorded in any position
Identifying live births			
SMR02	As above	Will identify live births occurring in hospital<br>SMR02 returns were enabled to cover home (as well as in hospital) births from Apr 2019, and coverage of home births should have been mandatory from Oct 2019, however technical difficulties mean that home births are still (as at July 2020) not recorded on SMR02 in most Boards	Condition on discharge=3 (delivered)<br>Outcome of pregnancy=1, 3, 4, 5 (live birth)
AND/OR<br>NRS live births	Record of statutory registration of a live birth (live born baby at any gestation)	Usually identifies all live births however statutory registration of live births was suspended from 23 March to 28 June 2020 inclusive when registrar offices closed<br>The only babies being registered during that period were those that subsequently die: this was done remotely along with the death registration to avoid parents having to register the birth in person later<br>A catch up programme of live birth registrations started on 29 June 2020	

AND/OR<br><br>NHS live birth notifications	Notification of live births from NHS Board maternity units to child health administration departments<br><br>This notification allows a record to be created for the child on the national child health information system: this in turn ensures the child is called for immunisations and child health reviews	As NRS live birth registration was suspended in March – June 2020 due to COVID-19 (see above), PHS has recently developed a new data extraction from the national child health information system of birth notification data<br><br>This will identify all live births known to NHS maternity services from Aug 2019 onwards<br><br>A small number of babies who die very soon after birth (before that day's notification data has been sent) will not be included as these babies do not need to be notified for ongoing care, however they will be covered by NRS registration as noted above	

It is possible that the same woman/pregnancy may have multiple records giving conflicting information on the outcome of the pregnancy.

In general, if any record indicates a termination of pregnancy, this should be taken as the outcome.

If an NRS stillbirth record is available for a baby but the corresponding SMR02 record indicates the baby was live born, this should be taken as a stillbirth.

The relevant gestation and date of event information in the various records, and how to deal with missing gestation information, is summarised below

National record	Gestation information available	Date of event information available	Dealing with missing gestation information (due to not recorded on that record, missing, or recorded but unfeasible)
SMR01	None	Date of admission<br>Date of discharge	Assume 12 weeks gestation at date of admission
SMR02	Gestation in completed weeks at end of pregnancy available on records where Condition on discharge=2 or 3 (aborted or delivered)	Date of admission<br>Date of discharge<br>Date of delivery on records where Condition on discharge=3 (delivered)	Miscarriage records with missing gestation, assume 12 weeks gestation at date of admission<br>ToP records with missing gestation (and not available from AAS), assume 16 weeks gestation at date of admission<br>Stillbirth delivery records with missing gestation (and not available from NRS), assume 32 weeks gestation at date of delivery<br>Live birth delivery records with missing gestation, assume 40 weeks gestation at date of delivery
NRS stillbirths	Gestation in completed weeks at date of stillbirth available	Date of stillbirth	Assume 32 weeks gestation at date of delivery (if not available from SMR02)
AAS	Gestation in completed weeks at date of termination available	Date of termination (date of administration of antiprogesterone for medical ToPs)	Assume 10 weeks gestation at date of termination (if not available from SMR02)
NRS live births	None	Date of birth	Assume 40 weeks gestation at date of birth (if not available from SMR02)

NHS live birth notifications	Gestation in completed weeks at date of birth available<br>(Although note this data has not been used before by PHS so will require checking before use)	Date of birth	Assume 40 weeks gestation at date of birth (if not available from SMR02)

The time lag inherent in the different data returns is summarised below

National record	Time lag inherent in data source
SMR01	Records should be returned to PHS within 6 weeks of patient's discharge (in practice sometimes longer)<br>Monthly batches (all records received to that point) are then uploaded to the analysis platform (SMRA) around the middle of each month<br>Records are CHI seeded as they are uploaded to the analysis platform (and CHI seeding is refreshed monthly thereafter to pick up any previously unseeded records)<br>CHI seeding usually complete on first attempt<br>So: records relating to discharges in Jan XX should be available for linkage and analysis within PHS in mid Apr XX (2.5 month lag)
SMR02	Records should be returned to PHS within 6 weeks of patient's discharge (in practice sometimes longer)<br>Monthly batches (all records received to that point) are then uploaded to the analysis platform around the middle of each month<br>Records are CHI seeded as they are uploaded to the analysis platform (and CHI seeding is refreshed monthly thereafter to pick up any previously unseeded records)<br>Maternal CHI seeding usually complete on first attempt<br>Baby CHI seeding usually complete on second attempt<br>So: as linkage of SMR02 records is generally through maternal CHI, records relating to discharges in Jan XX should be available for linkage and analysis within PHS in mid Apr XX (2.5 month lag)

NRS stillbirths	Registration required within 21 days of birth<br>Data transferred by NRS to PHS weekly<br>Monthly batches (stillbirths registered up to the end of the previous month) are then uploaded to the analysis platform around the middle of each month<br>In parallel, records are sent to NHSCR monthly for seeding of maternal CHI<br>As seeded records are returned from NHSCR, the CHIs are added to the records on the analysis platform<br>So: records relating to stillbirths occurring in Jan XX should be available for linkage and analysis within PHS in mid May XX (3.5 month lag)<br>(Note: almost all stillbirths will have an SMR02 record so can be identified and linked with 2.5 month lag)
AAS	Notification to CMO required within 7 days of termination<br>Records forwarded to PHS and entered into AAS system (includes automated CHI seeding) within 6 weeks of date of termination<br>So: records relating to terminations occurring in Jan XX should be available for linkage and analysis within PHS in mid Mar XX (1.5 month lag)

NRS live births	Registration required within 21 days of birth<br>Data transferred by NRS to PHS weekly<br>Monthly batches (live births registered up to the end of the previous month) are then uploaded to the analysis platform around the middle of each month<br>Records are seeded with baby CHI as they are uploaded to the analysis platform (and CHI seeding is refreshed monthly thereafter to pick up any previously unseeded records)<br>Baby CHI seeding usually complete on second attempt<br>In parallel, monthly batches are seeded with the mother's CHI by bespoke linkage to SMR02 after a 6 month lag (i.e. records for births in Jan XX and matched against SMR02 in Jul XX)<br>Records with no maternal CHI found are then matched against the full CHI database<br>Residual records with still no maternal CHI are then sent to NHSCR in monthly batches<br>So: as linkage of NRS live birth records generally requires both maternal and baby CHI (to allow intergenerational linkage), records relating to births in Jan XX should be available for linkage and analysis within PHS in mid Oct XX (8.5 month lag)<br>(Note: all live births from Aug 2019 onwards will have a birth notification record available so can be identified and linked with a 1 month lag)
NHS live birth notifications	Live births are notified to the NHS Board child health admin department within 1 working day of date of birth and are keyed into the national child health info system promptly (same or subsequent day)<br>PHS extracts notification data (including baby's CHI) from the national child health info system weekly<br>Maternal CHI is then seeded onto the data extracts weekly<br>So: records relating to births in Jan XX should be available for linkage and analysis within PHS in Feb XX (1 month lag)

National data sources identifying continuing pregnancies as early as possible

As part of the response to COVID-19, PHS has established a new national data return providing information on women booking for antenatal care. This will allow us to identify pregnant women before the end of their pregnancy, and hence monitor SARS-CoV-2 infections occurring in pregnant women in closer to real time. Further information on this data source is provided below.

Data items being requested in the new data feed include

- Maternal CHI
- Mother's Forename, Surname, Date of Birth, and Postcode in case CHI is missing and needs to be appended
- Date of Booking
- Gestation at booking
- Date of Last Menstrual Period (in case gestation is missing)

PHS has asked NHS Boards to provide an initial submission of historic data on all women booking from 1 April 2019, then subsequent weekly updates. The weekly updates will give information on women who have booked in the most recent week, and also update any records relating to the previous 2 weeks if those have changed since the previous submission. The current assumption is that this data will be submitted with maternal CHI complete, hence additional lag for CHI seeding will not be required but this is being kept under review.

This dataset will identify all women booking for NHS antenatal care. The method of providing booking services has changed in many areas due to COVID-19, with many Boards now providing the initial booking appointment remotely, with the woman subsequently attending in person for her initial ultrasound scan and blood tests[1]. To ensure that the dataset allows us to identify pregnant women as early as possible in their maternity care journey, the 'booking' event that is captured in the above dataset has therefore been defined as '*the date on which maternity services had the first planned/structured contact with a pregnant woman to assess her history and needs so that local maternity services can provide further care such as an early pregnancy scan and antenatal screening tests*', i.e. the initial remote contact.

[1] https://tec.scot/clinical-specialty-guidance/

Available national data shows that, pre-COVID-19, at least 90% of pregnant woman attended their booking appointment by 12^{+6} weeks gestation[2]. There is currently no evidence that gestation at initial booking has increased due to COVID-19. If gestation and LMP are both missing on antenatal booking records, we will therefore assume the woman was at 12 weeks gestation at the date of booking.

[2] https://www.isdscotland.org/Health-Topics/Maternity-and-Births/Publications/

Defining start and end date of pregnancies

For pregnancies that have ended

Pregnancy end dates will be taken from end of pregnancy records as noted above

Pregnancy start date (date of conception) will be imputed from the pregnancy end date and the gestation at pregnancy end – 2 weeks

For continuing pregnancies

Pregnancy start date (date of conception) will be imputed from the date of antenatal booking and the gestation at booking – 2 weeks, or from the date of last menstrual period + 2 weeks if gestation is missing and LMP is provided

Time lags inherent in data sources identifying COVID-19 status and relevant outcomes

In general, the time lags inherent in data sources identifying COVID-19 status and relevant outcomes are less than (or at least no more than) those inherent in the various data sources required to identify pregnancy status.

The only additional lag that needs to be considered is that seen in Scottish Birth Record (SBR) records. SBR records are not returned to PHS as such. Rather, PHS takes a monthly download of data held on the system for analysis purposes. In most NHS Boards, the SBR system is used to generate a CHI number for a baby shortly after birth. Skeleton records with minimal demographic data are therefore available for all babies in a timely manner. For babies admitted to neonatal care, clinical coding staff within NHS Board admin departments are responsible for completing additional variables within a baby's SBR record following their discharge. There is no national standard for when this should be done and in practice the lag between discharge and a completed record being available varies between Boards. Some Boards achieve broadly complete records within 3 months whereas others take considerably longer. Currently (June 2020) NHS Borders and NHS Dumfries & Galloway have not coded any SBR records (or provided comparable data directly to PHS) since June 2017 and April 2018 respectively. SBR data is therefore unlikely to provide a complete picture of neonatal admissions within the timeframes set out for this analysis (i.e. for babies born in March 2020, the data available to PHS on SBR by July 2020 will only provide a partial picture of admissions to neonatal care). PHS may explore getting a new national feed from NHS Boards of more real time data on neonatal admissions to mitigate this problem if feasible.
