# Supplementary material for: COVID-19 in Pregnancy in Scotland (COPS): protocol for an observational study using linked Scottish national data
Source: BMJ Open. 2020 Nov 26;10(11):e042813. doi: 10.1136/bmjopen-2020-042813 (PMC7691999; doi:10.1136/bmjopen-2020-042813)
Supplement: Author's manuscript [file bmjopen-2020-042813.draft_revisions.pdf]

BMJ Open

BMJ Open is committed to open peer review. As part of this commitment we make the peer review history of every article we publish publicly available.

When an article is published we post the peer reviewers' comments and the authors' responses online. We also post the versions of the paper that were used during peer review. These are the versions that the peer review comments apply to.

The versions of the paper that follow are the versions that were submitted during the peer review process. They are not the versions of record or the final published versions. They should not be cited or distributed as the published version of this manuscript.

BMJ Open is an open access journal and the full, final, typeset and author-corrected version of record of the manuscript is available on our site with no access controls, subscription charges or pay-per-view fees (http://bmjopen.bmj.com).

If you have any questions on BMJ Open's open peer review process please email info.bmjopen@bmj.com

BMJ Open

COVID-19 in Pregnancy in Scotland (COPS): protocol for an observational study using linked Scottish national data

Journal:	*BMJ Open*
Manuscript ID	bmjopen-2020-042813
Article Type:	Protocol
Date Submitted by the Author:	18-Jul-2020
Complete List of Authors:	Stock, Sarah; The University of Edinburgh, Usher Institute; The University of Edinburgh MRC Centre for Reproductive Health, Tommy's Centre for Maternal and Fetal Health<br>McAllister, David; University of Glasgow; Public Health Scotland, David McAllister<br>Vasileiou, Eleftheria; The University of Edinburgh, Usher Institute;<br>Simpson, Colin; Victoria University of Wellington,<br>Stagg, Helen R.; University of Edinburgh, Usher Institute<br>Agrawal, Utkarsh; University of St Andrews, School of Medicine<br>McCowan, Colin ; University of St. Andrews<br>Hopkins, Leanne; Public Health Scotland<br>Donaghy, Jack; Public Health Scotland<br>Ritchie, Lewis; Aberdeen University, General Practice and Primary Care<br>Robertson, Chris; University of Strathclyde, Department of Mathematics and Statistics<br>Sheikh, Aziz; University of Edinburgh, Division of Community Health Sciences<br>Wood, Rachael; NHS National Services Scotland, Information Services Division; University of Edinburgh, Child Life and Health
Keywords:	COVID-19, Epidemiology < INFECTIOUS DISEASES, OBSTETRICS, NEONATOLOGY, PERINATOLOGY

SCHOLARONE™
Manuscripts

BMJ

I, the Submitting Author has the right to grant and does grant on behalf of all authors of the Work (as defined in the below author licence), an exclusive licence and/or a non-exclusive licence for contributions from authors who are: i) UK Crown employees; ii) where BMJ has agreed a CC-BY licence shall apply, and/or iii) in accordance with the terms applicable for US Federal Government officers or employees acting as part of their official duties; on a worldwide, perpetual, irrevocable, royalty-free basis to BMJ Publishing Group Ltd ("BMJ") its licensees and where the relevant Journal is co-owned by BMJ to the co-owners of the Journal, to publish the Work in this journal and any other BMJ products and to exploit all rights, as set out in our licence.

The Submitting Author accepts and understands that any supply made under these terms is made by BMJ to the Submitting Author unless you are acting as an employee on behalf of your employer or a postgraduate student of an affiliated institution which is paying any applicable article publishing charge ("APC") for Open Access articles. Where the Submitting Author wishes to make the Work available on an Open Access basis (and intends to pay the relevant APC), the terms of reuse of such Open Access shall be governed by a Creative Commons licence – details of these licences and which Creative Commons licence will apply to this Work are set out in our licence referred to above.

Other than as permitted in any relevant BMJ Author's Self Archiving Policies, I confirm this Work has not been accepted for publication elsewhere, is not being considered for publication elsewhere and does not duplicate material already published. I confirm all authors consent to publication of this Work and authorise the granting of this licence.

For peer review only

COVID-19 in Pregnancy in Scotland (COPS): protocol for an observational study using linked Scottish national data

Sarah J Stock, Usher Institute, University of Edinburgh NINE Edinburgh BioQuarter, 9 Little France Road, Edinburgh EH16 4UX UK sarah.stock@ed.ac.uk +44 (0)7894629934 (corresponding author) ORCID ID 0000-0003-4308-856X

David McAllister, Public Health Scotland, UK. University of Glasgow, Glasgow, UK, ORCID ID 0000-0003-3550-1764

Eleftheria Vasileiou, Usher Institute, University of Edinburgh, Edinburgh, UK ORCID ID 0000-0001-6850-7578

Colin R Simpson, School of Health, Wellington Faculty of Health, Victoria University of Wellington, Wellington, New Zealand; Usher Institute, University of Edinburgh, Edinburgh, UK ORCID ID 0000-0002-5194-8083

Helen R Stagg, Usher Institute, University of Edinburgh, Edinburgh, UK ORCID ID 0000-0003-4022-3447

Utkarsh Agrawal, School of Medicine, University of St Andrews, St Andrews, UK ORCID ID 0000-0001-5181-6120

Colin McCowan, School of Medicine, University of St Andrews, St Andrews, UK ORCID ID 0000-0002-9466-833X

Leanne Hopkins, Public Health Scotland, UK ORCID ID 0000-0002-7487-4363

Jack Donaghy, Public Health Scotland, UK ORCID ID 0000-0002-6137-1601

For peer review only

Lewis Ritchie, Institute of Applied Health Sciences, University of Aberdeen, UK ORCID ID 0000-0002-9380-7641

Chris Robertson, Public Health Scotland, UK. Department of Mathematics and Statistics, University of Strathclyde, Glasgow, UK ORCID ID 0000-0001-6848-5241

Aziz Sheikh, Usher Institute, University of Edinburgh, Edinburgh, UK ORCID ID 0000-0001-7022-3056

Dr Rachael Wood, Public Health Scotland and University of Edinburgh, UK ORCID ID 0000-0003-4453-623X

Key Words: COVID-19, Pregnancy, Maternal, Neonatal, Perinatal, Coronavirus

Word Count: 5050

For peer review only

For peer review only - http://bmjopen.bmj.com/site/about/guidelines.xhtml

ABSTRACT

Introduction

The effects of SARS-CoV-2 in pregnancy not fully delineated. We will describe the incidence of COVID-19 in pregnancy at population level in Scotland, in a prospective cohort study using linked data. We will determine associations between COVID-19 and adverse pregnancy, neonatal and maternal outcomes; and the proportion of confirmed cases of SARS-CoV-2 infection in neonates associated with maternal COVID-19.

Methods and analysis

Prospective cohort study using national linked datasets. We will include all women in Scotland, UK, who were pregnant on, or became pregnant after, 1st March 2020 (the date of the first confirmed case of SARS-CoV-2 infection in Scotland), and all births in Scotland from 1st March 2020 onwards. Individual-level data will be extracted from datasets containing details of all livebirths, stillbirth, terminations of pregnancy, and miscarriages and ectopic pregnancies treated in hospital or attending general practice. Records will be linked within the Early Pandemic Evaluation and Enhanced Surveillance of COVID-19 (EAVE II) platform, which includes primary care records, virology and serology results, and details of COVID-19 Community Hubs and Assessment Centre contacts and deaths. We will perform analyses using definitions for confirmed, probable and possible COVID-19, and report serology results (where available). Outcomes will include congenital anomaly, miscarriage, stillbirth, termination of pregnancy, preterm birth, neonatal infection, severe maternal disease and maternal deaths. We will perform descriptive analyses and appropriate modelling, adjusting for demographic and pregnancy characteristics, and the presence of co-morbidities. The cohort will provide a platform for future studies of the effectiveness and safety of

For peer review only - http://bmjopen.bmj.com/site/about/guidelines.xhtml

therapeutic interventions and immunisations for COVID-19, and their effects on childhood and developmental outcomes.

Ethics and dissemination

COPS is a sub-study of EAVE II, which has approval from the National Research Ethics Service Committee. Findings will be reported to Scottish Government, Public Health Scotland and published in peer reviewed journals.

For peer review only

For peer review only - http://bmjopen.bmj.com/site/about/guidelines.xhtml

ARTICLE SUMMARY

Strengths and limitations of this study

- We will interrogate Scottish national data at the population level to provide information on the incidence of, and outcomes following, COVID-19 outcomes in pregnant women.
- We are expanding an existing national pandemic reporting platform (EAVE II) to include assessment of all pregnancy outcomes. EAVE II uses de-identified individual patient-level data for almost the entire population of Scotland from general practices, hospitals, death registry, virology (Reverse Transcriptase Polymerase Chain Reaction; RT-PCR) and serology tests to investigate the epidemiology of COVID-19.
- This is an observational study and residual confounding is a potential concern.

For peer review only

INTRODUCTION

The effects of novel Severe Acute Respiratory Syndrome Coronavirus -2 (SARS-CoV-2) in pregnancy are yet to be fully delineated.[1] Pregnant women are at greater risk of complications and severe disease from infection with other coronaviruses including SARS and Middle Eastern Respiratory Syndrome (MERS).[2 3] Pregnant women were thus identified as a potential vulnerable group in some countries and advised to take additional precautions as the Coronavirus Disease 2019 (COVID-19) pandemic unfolded.[2 3 4]

To inform public health policy, it is crucial to determine the effects of SARS-CoV-2 infection on maternal, pregnancy, and neonatal health. SARS-CoV-2 transmission from mother to baby (antenatally or intrapartum) appears to be possible,[5] but the proportion of pregnancies affected and the clinical significance is uncertain. Potential effects of the virus on miscarriage, congenital anomalies, fetal growth, timing of delivery, and stillbirth are unknown. We know from other viral infections in pregnancy that infections with mild maternal symptomatology can have substantial impacts on the developing fetus (e.g. Cytomegalovirus, Parvovirus, Zika virus),[6] although mechanisms of placental transmission of virus vary.[7] The canonical receptors for SARS-CoV-2 (the angiotensin- converting enzyme 2 [ACE2] receptor and the serine protease TMPRSS2) are not co-expressed in the placenta, making placental infection unlikely.[8 9] Nevertheless, case reports have shown evidence suggestive of viral infection of the placenta, in association with pregnancy complications such as pre-eclampsia and abruption[10] and second trimester miscarriage.[11] Reports that include neonatal test results for SARS-CoV-2 show positive cases only in a minority of babies, with significant respiratory disease being rare in neonates.[12] However, some babies born to mothers who had COVID-19 have increased concentrations of both immunoglobulin IgM and IgG for SARS-CoV-2.[13 14] As IgM cannot cross the placenta, neonatal circulating

For peer review only - http://bmjopen.bmj.com/site/about/guidelines.xhtml

SARS-CoV-2 IgM indicates vertical transmission of virus, although all the infants in reports so far have been asymptomatic and tested negative for SARS-CoV-2 viral RNA at birth.[13 14] There are also plausible links between SARS-CoV-2 and pregnancy complications such as preterm birth, which may be mediated either as a manifestation of COVID-19 disease itself; or indirectly through increased stress due to the pandemic and containment measures, or through altered physician threshold for iatrogenic preterm delivery in women with infection.[1]

Understanding the effects of COVD-19 at different stages in pregnancy and perinatally will help inform policy on shielding strategies, and advice to pregnant women and those considering pregnancy. It is also essential to inform immunisation strategies when vaccines are available. For example, immunisation in early pregnancy may help protect against maternal infection during pregnancy and reduce complications; but immunisation in later pregnancy may be preferential to provide passive immunisation to babies if neonatal infection is the predominant concern.

There are a number of surveillance studies gathering data on pregnant women with COVID-19 currently underway in the UK, summarised in Table 1.

Collectively, these surveillance studies can provide detailed characterisation of selected groups of pregnant women (and neonates) affected by COVID-19. The study outlined in this protocol will complement these existing studies by providing population-based information (for the whole of Scotland) on the risks of, and outcomes following, COVID-19 at any stage of pregnancy for women in the community and/or admitted to hospital.

The primary objectives of the COVID-19 in Pregnancy in Scotland (COPS) study are to:

a) describe the incidence of SARS-CoV-2 infection and COVID-19, in the pregnant population;

For peer review only

b) determine associations between COVID-19 and adverse maternal, pregnancy, and neonatal outcomes;

c) determine the proportion of neonates with confirmed SARS-CoV-2 infection that are associated with COVID-19 in the baby's mother

Secondary objectives are to:

a) assess the proportion of COVID-19 cases in pregnant women and neonates who are included in relevant other enhanced surveillance studies (e.g. BPSU, CO-CIN);[15-17]

b) provide a platform to assess the safety and effectiveness of any new or existing prophylactic or therapeutic interventions (e.g. new or repurposed therapies, vaccines, antimicrobials) in pregnant women and their babies;

c) enable evaluation of the longer-term sequelae of maternal SARS-CoV-2, and therapeutic interventions to mitigate SARS-CoV-2 in pregnancy and in children.

This COPS study is a sub-study of the EAVE II study (Early Pandemic Evaluation and Enhanced Surveillance of COVID-19 observational study using linked Scottish national data).[18-20] EAVE II is a national, real-time, data platform to identify the population groups most at risk from SARS-CoV-2 infection and COVID-19 disease and mortality, linking Scottish General Practice (GP) records (5.4 million registered patients) with secondary care and laboratory datasets.[18] Pregnancy will be assessed as one of these at-risk groups. Within this COPS study protocol, we specify in detail the national datasets that will be incorporated within the EAVE II platform to enable pregnant women (and associated pregnancy start and end dates) to be reliably identified. We also specify in detail the maternal, pregnancy and neonatal outcomes following maternal COVID-19 that will be examined.

For peer review only

METHODS

Study design and population

This is a prospective cohort study using national maternity, community, hospital and laboratory linked datasets, in Scotland, UK. We will include all women in Scotland who were pregnant on, or became pregnant after 1st March 2020 (the date of the first confirmed case of SARS-CoV-2 infection in Scotland[21]), and all live born babies born in Scotland from 1st March 2020 onwards. The end date for the study will be determined by the future development, and in particular suppression of, the pandemic in Scotland.

Women in the cohort with the earliest dates of conception will only have been at risk of COVID-19 at the very end of their pregnancy. Women with more recent dates of conception will have been at risk for longer, up to women with date of conception from 1 March 2020 onwards who will be at risk from conception onwards (until viral transmission is completely suppressed).

We aim to use a dynamic cohort of the entire pregnant population; therefore, selection bias is not anticipated and the dataset will be fully generalisable to Scotland (with extensive generalisability to other high-income nations).

Databases

Individual-level data will be extracted from the datasets listed below. Records relating to the same individual will be linked deterministically using the Community Health Index (CHI) number.[22] The CHI number is a unique identifier provided by National Health Service (NHS) Scotland for each resident registered with a general practice. Public Health Scotland routinely adds both maternal and baby CHI numbers to live birth registration records: these 'spine'

For peer review only - http://bmjopen.bmj.com/site/about/guidelines.xhtml

records will be used to facilitate intergenerational linkage of records relating to mothers and their babies.

An overall schema for the planned linkage is provided in Figure 1. Unless otherwise specified, included datasets are held by Public Health Scotland (PHS) and PHS is the data controller.

Datasets to identify pregnant women in the general population and associated pregnancy start and end dates, and pregnancy and neonatal outcomes

i) Antenatal booking records: This is a new national data return developed as part of the response to the COVID-19 pandemic providing information on all women booking for antenatal care with NHS maternity services throughout Scotland. It will be used for identification of women with ongoing pregnancies in near real-time. All other records identify end of pregnancy events, thus there is a time-lag before records are generated. More than 99% of births in Scotland book for antenatal care with NHS maternity services.[23]

ii) Abortion Act Scotland [AAS] records: These are statutory notifications of termination of pregnancy, and will be used to identify all terminations of pregnancy, and including terminations of pregnancy indicated by congenital anomaly.[24]

iii) National Records of Scotland (NRS) statutory stillbirth registrations: Scottish legislation requires all stillbirths at 24 weeks gestation or more to be registered with NRS.[25]

iv) NRS statutory live birth registrations: Scottish legislation requires all live births at any gestation to be registered with NRS.[26]

v) NHS Scottish health board live births: A new national data return developed as part of the response to the COVID-19 pandemic (specifically to mitigate unavailability of NRS statutory live birth registration records when registration processes were suspended) providing

For peer review only - http://bmjopen.bmj.com/site/about/guidelines.xhtml

information on all live births notified by maternity services to NHS Board child health administrative departments.

vi) GP data: Data from all patients registered in general practices are included in the EAVE II platform.[18] In COPS, these records will be used for identification of women with early miscarriage or ectopic pregnancy not managed in hospitals, and potential confounding co-morbidities.

vii) Scottish Morbidity Record (SMR) 01: The SMR01 database includes all general day case and in-patient admissions in Scotland.[27] Admissions to neonatal, maternity, and mental health care are excluded from SMR01 as they are covered by other specialist datasets. SMR01 records are included in the EAVE II platform, and in COPS will be used for identification of women with early miscarriage or ectopic pregnancy managed in hospitals, and potential confounding co-morbidities.

viii) SMR 02: The SMR02 database includes all day case and in-patient admissions to maternity specialties in Scotland.[27] It will be used for identification of later miscarriage, stillbirth, and live births managed in maternity units (≥98% of births in Scotland) and some home births (≤2% of births in Scotland).[23]

ix) Scottish Birth Record (SBR): The SBR records basic demographic data on all births in Scotland, and additional clinical information and diagnostic and operational procedure codes on babies admitted to neonatal care.[28] It will be used to identify neonates admitted to neonatal care.

x) Scottish Intensive Care Society Audit Group (SICSAG) records: This is a national database of patients admitted to adult general critical care units in Scotland detailing information on the management of critically ill or injured patients. All general Intensive Care

For peer review only - http://bmjopen.bmj.com/site/about/guidelines.xhtml

Units and combined ICU/High Dependency Units (HDU) collect data and more than 90% of general High Dependency Units and a number of specialist ICU and HDUs also provide records.[29] In COPS these will be used to identify women admitted to critical care with COVID-19.

Datasets to identify women with confirmed SARS-CoV-2 infection or COVID-19

i) Electronic Communication of Surveillance in Scotland (ECOSS)[30] and other viral RT-PCR and serology results held separately by PHS are included on the EAVE II platform.[18] In COPS, these will be used for identification of pregnant women and neonates with positive viral RT-PCR and serology, and women with negative viral RT-PCR.

ECOSS is a database that holds surveillance data on various microorganisms (e.g. influenza virus, coronavirus) and infections reported from NHS diagnostic and reference laboratories.[30] Data on laboratory results for all SARS-CoV-2 RT-PCR tests carried out in Scotland are being collated by ECOSS and can be linked to other data sources.[30]

In sub-studies, residual sera from routine antenatal booking blood tests and 28 week gestation blood group and red cell antibody screen samples will be tested for SARS-CoV-2 antibodies. Residual sera from other blood tests conducted as part of routine (not COVID-19 related) primary and secondary care, and blood donation, are also being tested for SARS-CoV-2 antibodies as part of the surveillance of the pandemic in Scotland, and any results relating to pregnant women will also be incorporated.[31] Results from these sub-studies will be linked to pregnancy records and used to determine exposure to SARS-CoV-2 by the presence of antibodies.[18]

ii) GP consultations, GP out of hours attendances, NHS24 calls, COVID-19 phone assessment hub calls, and COVID-19 clinical assessment centre attendances are linked on the

For peer review only - http://bmjopen.bmj.com/site/about/guidelines.xhtml

EAVE II platform.[18] We will extract data on pregnant women with possible COVID-19 from GP records and a network of COVID-19 Community Hubs and Assessment Centres established by NHS Health Boards across Scotland. These provide a direct and rapid route for people with COVID-19 symptoms that have worsened or not improved after a week to seek advice and primary care. The pathway for management of patients in the community with symptoms suggestive of COVID-19 has evolved as the pandemic has progressed. In early March 2020, patients with symptoms were advised to phone their GP in hours or NHS 24 out of hours for advice. Patients requiring face to face assessment were then seen in GP surgeries or GP out of hours centres. On 17 March 2020, the Scottish Government published details of a new national patient pathway, whereby all patients with symptoms (in or out of hours) were encouraged call NHS 24 as the initial point of contact.[32] Patients thought likely to have COVID-19 were then passed to a new, dedicated NHS24 COVID-19 phone assessment hub. Those requiring face-to-face assessment in general were then seen in (or visited at home by staff from) an NHS24 COVID-19 clinical assessment centre, although pregnant women could alternatively be directed to their maternity service triage base.[32]

iii) Unscheduled Care Datamart (UCD): This links data from NHS 24, Scottish Ambulance Service, Out of Hours Primary Care, Emergency Department, Acute and Mental Health admissions and Deaths to show a Continuous Unscheduled Care Pathway. Data is included in the EAVE II platform.[18] In COPS, we will use Scottish Ambulance Service incident and A&E attendance records for identification of women with possible COVID-19.[33]

iv) NRS statutory death registrations will be used for identification of any women with COVID-19 recorded as cause of death.[34]

v) SMR01, SMR02, and NRS stillbirths will be used to identify women with COVID-19 recorded as cause of admission/stillbirth.[25 27]

For peer review only - http://bmjopen.bmj.com/site/about/guidelines.xhtml

Datasets recording treatments, vaccination, shielding status and inclusion in other studies

i) Prescribing Information System (PIS). This includes information on all prescribed medications that are dispensed in the community in Scotland.[35] Lookback records are included in the EAVE II platform[18] and used to provide information on the presence of co-morbidities. In COPS records will also be used provide information on COVID-19 treatments given.

ii) Scottish Hospital Electronic Prescribing and Medicines Administration (HEPMA) systems are currently available within four Scottish hospitals, and data from these are linked in the EAVE II platform[18] to provide data on medications for COVID-19 administered in hospitals.[36]

iii) GP consultation records included on the EAVE II platform[18] will be used to extract information on vaccinations for SARS-CoV-2 and other viruses.

iv) We will identify pregnant women and neonates with COVID-19 in existing enhanced surveillance studies using minimal 'flag' variables from the BPSU and CO-CIN studies,[15-17] as well as other trials who can provide this data.

Exposure and Outcome definitions

Pregnancy, start and end dates and pregnancy outcomes

A pregnancy will be defined by presence of a record pertaining to a pregnancy. As well as identifying completed pregnancies, through a record of any pregnancy outcome, we will be able to identify ongoing pregnancies at population level, through inclusion of records from women booked for antenatal care.

For peer review only

The following definitions will be used for pregnancy outcomes. The codes and data sources used to identify relevant records, are described in detail in Supplementary material.

i) Ectopic pregnancy: This will include any early pregnancy loss where the pregnancy is implanted outwith the uterus

ii) Spontaneous pregnancy losses: These will be defined as miscarriage at less than 20 weeks gestation, late fetal losses at 20 - 23 weeks if there are no signs of life; and stillbirth if birth occurs at 24 weeks' gestation or more and there are no signs of life. If numbers allow, the miscarriages will be further split into the more clinically meaningful outcome categories of miscarriage at less than 14 weeks gestation; and miscarriage 14 weeks gestation and beyond.

iii) Termination of pregnancy: These will be subclassified by the grounds for termination of pregnancy of the Abortion Act 1967.[37]

iv) Live birth: The birth of a baby at any gestation with signs of life. No lower gestational limit will be used although in practice around 22 weeks gestation would be considered the lower limit at which live born babies may survive

Pregnancy start date will be taken as the date of conception. In pregnancies that have ended date of conception will be imputed from

"Date of conception = pregnancy end date - (the number of weeks of gestation at pregnancy end + 2 weeks)"

In ongoing pregnancies that have booked with maternity services and have a documented estimated date of delivery (EDD), date of conception will be imputed from

"Date of conception = date of antenatal booking – (gestation at booking + 2 week)"

For peer review only - http://bmjopen.bmj.com/site/about/guidelines.xhtml

In ongoing pregnancies without a documented EDD, date of conception will be imputed from "Date of conception = date of last menstrual period + 2 weeks".

It is standard care for women who book with maternity services in Scotland have a first trimester ultrasound scan (usually 11-13+6 weeks gestation) to determine EDD, from which gestation is calculated. Booking takes place around 10 weeks gestation.

SARS-CoV-2 infection and COVID-19

We will use the following definition for COVID-19.

- Confirmed COVID-19 in pregnancy will be defined as positive viral PCR for SARS-CoV-2 on a test.
- Probable COVID-19 will be defined as COVID-19 recorded on a hospital admission, stillbirth, or a maternal death record (using ICD10 codes U07.1, U07.2, B34.2, B97.2)
- Possible COVID-19 will be defined as meeting one or more of the following criteria:
    - GP consultation or GP out of hours attendance coded as possible COVID-19
    - NHS24 call coded as possible COVID-19
    - Patient triaged to NHS24 COVID-19 phone assessment hub or clinical assessment centre
    - Scottish Ambulance Service call for possible COVID-19
    - A&E attendance coded as possible COVID-19
    - Negative SARS-CoV-2 viral PCR test when the test was taken for clinical indications (i.e. excluding tests taken for routine testing of asymptomatic individuals)

For peer review only - http://bmjopen.bmj.com/site/about/guidelines.xhtml

The above definitions are hierarchical, e.g. a positive SARS-CoV-2 nucleic acid test assigns a woman to the confirmed COVID-19 group, regardless of the presence of other records.

SARS-CoV-2 infections during pregnancy will be identified if the event of interest (e.g. SARS-CoV-2 nucleic acid test taken, admission, unscheduled care attendance) occurs between 14 days prior to the estimated date of conception (to include women who could be viraemic periconceptually) and the end of pregnancy.

We will seek to access serological data as these become available. We will report the proportion of women with circulating IgG and/or IgM for SARS-CoV-2 and may incorporate serology results in case definitions and/or use in additional analyses as data mature. The timing of exposure to/infection with SARS-CoV-2 is more difficult to ascertain from serology results than from the other indicators of (possible) infection listed above. Dates of serological testing; start and end of pregnancy; and plausible start (and, if applicable, end) of transmission of SARS-CoV-2 in Scotland will be taken into account when identifying women with exposure before, during, and after pregnancy. Seroconversion windows will also be considered for women with sequential serology results.

Other diagnostic and exposure categories may be added as the pandemic develops and diagnostic criteria change.

Fetal and neonatal outcomes

The following fetal and neonatal outcomes will be included.

For peer review only

i) Congenital anomaly (major structural anomaly as defined by EUROCAT [38] diagnosed in any pregnancy terminated at any gestation due to anomaly; miscarriage or stillbirth at ≥20 weeks; or live born baby diagnosed at <28 days of age)

ii) Preterm birth (<37 weeks) categorised as spontaneous or medically indicated (i.e. following induction of labour or elective Caesarean section undertaken to mitigate clinical risk)

iii) Small for gestational age (birthweight <10th centile by WHO-UK90 growth reference [39])

iv) Admission to neonatal care

v) Neonatal SARS-CoV-2 infection (currently defined as positive viral RT-PCR test on sample taken from baby aged 0-27 days, definition may be expanded to include results of serology tests as evidence and testing options accumulate)

vi) Neonatal mortality (death of a live born baby at <28 days of age)

vii) Extended perinatal mortality (stillbirth or neonatal mortality)

Maternal outcomes

We will collect data on the following maternal outcomes:

- COVID-19 disease requiring any hospital admission (defined as a patient admitted within 14 days of confirmed or probable COVID-19, or with confirmed or probable COVID-19 during admission)
- Severe COVID-19 disease requiring critical care admission or resulting in death (defined as patient admitted to critical care or dying within 28 days of confirmed or

For peer review only

probable COVID-19, or with confirmed or probable COVID-19 during hospital admission, regardless of recorded cause of death)

- Any maternal death (defined as the death of a woman while pregnant or within 42 days of the termination of pregnancy, irrespective of the duration and site of the pregnancy, from any cause related to or aggravated by the pregnancy or its management but not from accidental or incidental causes)

Population characteristics and confounding factors

A number of maternal and pregnancy characteristics will be collected that could be potential confounders or effect modifiers.

i) Demographics including age band and socioeconomic status determined by the Scottish Index of Multiple Deprivation (SIMD) classification of material deprivation.[40] SIMD Quintiles 1 through 5 refer to the small geographical areas (data zones) each containing 20% of the Scottish population, with quintile 1 indicating the most deprived areas. The SIMD is a combination of 38 indicators of the following seven domains: income, employment, health, education, housing, geographical access to services, and crime. We will also include urban/rural status of maternal residence based on the urban/rural 8 fold classification (UR8)[41] where 1 is assigned to large urban areas and 8 is assigned to remote rural areas. We recognise ethnicity to be a complex indicator variable related to sociodemographic factors, health systems use, pregnancy and health outcomes and genetics. We will explore the possibility of including self-reported maternal ethnicity although missing data may preclude this.

ii) Clinical at-risk groups of individuals with certain underlying medical conditions thought to increase risk of COVID-related complications. The following clinical at-risk conditions are identified in the EAVE II platform[18]: a) chronic respiratory disease; b) chronic heart disease;

For peer review only - http://bmjopen.bmj.com/site/about/guidelines.xhtml

c) chronic liver disease; d) chronic kidney disease; e) chronic liver disease; f) chronic neurological disease; g) Diabetes; h) Conditions or medications causing impaired immune function; i) Asplenia or dysfunction of spleen and j) body mass index (BMI). In addition, we will link pregnancy records with the shielded patient list included in the EAVE II platform (those with extremely high risk of severe manifestation of SARS-CoV-2 infection, and hence advised by Scottish Government to 'shield' during the pandemic). We will re-categorise clinical risks for the pregnant population as i) Diabetes (Type I; Type II; Other pre-pregnancy; Gestational Diabetes); ii) clinically vulnerable risk group (for whom seasonal influenza vaccination is recommended outwith pregnancy); iii) clinically extremely vulnerable risk group (those advised to 'shield' during the pandemic);[42] and iv) no clinical at-risk condition. BMI, which can be associated with adverse pregnancy outcomes as well as COVID-disease will be included separately, with pre-pregnancy BMI or BMI at antenatal booking categorised as Underweight, Normal, Overweight, Obese and Severely Obese according to WHO definitions.[43] Other categorisation will be considered depending on numbers of pregnant women with these conditions, and emergence of patterns of risk for COVID-19 disease.

iii) Smoking status in pregnancy will be presented into the following four categories: Current smoker, non-smoker, ex-smoker and not recorded. Smoking status will be taken from booking record and/or GP records.

iv) Obstetric characteristics (SMR02) will include previous pregnancies, plurality (number of babies), drug and alcohol use, antenatal steroid administration, mode of birth or management of pregnancy loss.

v) Clustering of outcomes by the 14 different Maternal NHS Board areas of residence.

For peer review only

Statistical analysis

General Approach

Baseline characteristics of all study participants will be described in relation to presence and absence of confirmed, probable or possible COVID-19 and outcomes of interest. Mean, median and proportions, together with a measure of dispersion will be provided where appropriate to describe differences between the various groups of interest based on the nature of each variable. Missing data will be provided for each variable. Two-tailed hypotheses tests will be used for all study's outcomes, with 95% confidence intervals presented to show precision of estimates, and p values reported. All analyses will be carried out using the R statistical programming language. We do not propose to make any formal statistical adjustment for the multiple comparisons. However, a caveat will be clearly expressed regarding the risks of over interpreting these data, given the multiple outcomes used. The approach to imputing estimated date of conception when gestation is missing on records indicating pregnancy status is detailed in Supplementary material 1. Missing data are otherwise not anticipated to be a substantial problem (and hence imputation techniques are not anticipated) but this will be confirmed once initial data extracts are available, and our approach to handling missing data will be confirmed prior to analysis.

Analyses will be updated monthly, providing results for sequential months, and also information on the cumulative risk of COVID-19 as women progress through their pregnancies. Simple smoothing techniques such as rolling averages will be used to facilitate presentation and interpretation of findings.

If/when numbers of cases allow, we will examine incidence of COVID-19, and report outcomes, in subgroups including by maternal age band, SIMD deprivation quintile, maternal

For peer review only

NHS Board area of residence and maternal comorbidity status. We may assess whether findings are robust to more stringent or emerging definitions of confirmed and suspected infection, in sensitivity analyses. Other sensitivity and subgroup analyses may be indicated by initial findings. We will clearly state which analyses were pre-specified and which were post-hoc.

Incidence of SARS-CoV-2 and COVID-19 in the pregnant population

We will perform descriptive analysis of the number of cases over the total number of pregnancies i.e. how many pregnant women have had confirmed, probable or possible COVID-19/ total number of pregnant women. Where timing of infection is known, we will describe incidence of SARS-CoV-2 infection by trimester of exposure - first trimester (0-13 weeks gestation) second trimester (14-27 weeks gestation); third trimester (≥28 weeks gestation); with denominators consisting of ongoing pregnancies in each trimester.

Associations between COVID-19 and adverse pregnancy, neonatal and maternal outcomes.

Initially we will perform a descriptive analysis comparing pregnancy outcomes in women with and without i) confirmed; ii) probable and iii) possible COVID-19. In order to create appropriate comparison groups, for each woman with COVID-19, we will identify ten women without COVID-19, with an ongoing pregnancy matched on gestation of diagnosis, and additionally matched on maternal age and maternal deprivation level. We will explore the need to match additional parameters such as NHS Board.

Occurrence of the outcomes of interest will be compared in women with and without COVID-19 using simple descriptive statistics (e.g. 95% confidence interval for the difference

For peer review only - http://bmjopen.bmj.com/site/about/guidelines.xhtml

in proportions, generated using methods which accommodate proportions close to zero) and visualised appropriately.

If/when sufficient cases of COVID-19 among pregnant women accrue, and the univariable comparisons described above suggest that outcomes differ between women with and without SARS-2-CoV or COVID-19, formal modelling will be undertaken to quantify the impact of infection on outcomes, adjusting appropriately for confounding. We will use direct acyclical graphs (DAGs) to identify which factors to adjust for to mitigate for confounding. Appropriate methods that accommodate the competing risk and time to event nature of pregnancy outcomes (example event history analysis and/or multistate modelling) will be used.

Association of SARS-CoV-2 in neonates with maternal COVID-19

We will use summary statistics to describe neonatal SARS-CoV-2 (currently defined as positive viral PCR for SARS-CoV-2 on sample taken from a baby aged 0-27 days old) by presentation of COVID-19 in the mother in different time periods (apparent onset of maternal illness >14 days prior to birth; 14 days prior to birth - date of birth; day 1 - 13 following birth; day 14 - 27 following birth).

Proportion of pregnant women and neonates with COVID-19 or SARS-CoV-2 that are included in relevant other enhanced surveillance studies (BPSU, CO-CIN)

We will use summary statistics to describe the number and proportion of cases included in the external surveillance studies, and any factors associated with inclusion e.g. hospital admission status and NHS Board area of residence.

For peer review only - http://bmjopen.bmj.com/site/about/guidelines.xhtml

Creation of a platform to assess the safety and effectiveness of any new or existing prophylactic or therapeutic interventions and assessment of childhood outcomes after pregnancy exposure to COVID-19

We will use summary statistics to describe the treatments and prophylactic interventions used in pregnancy. We plan future linkage of data within COPS with child health and education data to allow assessment of long-term outcomes.

Sample size

There are approximately 50,000 live births in Scotland per year, 13,000 terminations of pregnancy, 5,000 miscarriages managed in hospital and 200 stillbirths. The estimated number of women in the population who are pregnant at any one time is approximately 42,000.

We cannot influence the number of women with confirmed, probable or possible COVID-19 available for analysis hence sample size calculations will not be performed. We will report the precision with which we are able to estimate any association between COVID-19 and the outcomes of interest using confidence intervals as appropriate. An approximate estimate of the expected number of confirmed COVID-19 cases in pregnant women from March to May 2020 is presented in Table 2. It is likely that there will be further confirmed or probable cases in pregnant women identified through PCR testing processed through UK Government laboratories and clinical diagnoses on discharge (or possibly stillbirth or maternal death) records. In addition, it is likely that there will be considerably more possible cases among pregnant women based on the range of data sources listed above.

ETHICS AND DISSEMINATION

For peer review only

For peer review only - http://bmjopen.bmj.com/site/about/guidelines.xhtml

COPS is a sub-study of EAVE II, using unconsented data, which is covered by National Research Ethics Service Committee, South East Scotland 02 approval reference REC 12/SS/0201: SA 1 and Public Benefit and Privacy Panel approval reference 1920-0279. Public Health Scotland and the Chief Medical Officer for Scotland are both (independent) data controllers for the national AAS database of termination of pregnancy notifications, thus the Chief Medical Officer has been informed of the intended use of AAS records for this study.

The results of monthly analyses summarising the incidence of COVID-19 in pregnant women, and outcomes seen in women with COVID-19 and pregnant controls, will be reported through the Public Health Scotland COVID-19 enhanced surveillance cell to the Scottish Government's Chief Medical Officer's COVID-19 Advisory Group. Any results of formal modelling of outcomes that is undertaken will be reported through the same route. Results reported through this route may be provided as management information (i.e. without application of statistical disclosure control restrictions) as appropriate.

Results will also be submitted for peer reviewed academic publication and presented at international conferences. All results put into the public domain will be subject to statistical disclosure control according to usual Public Health Scotland processes. Meta-data produced in this study will also become available to Health Data Research UK (HDRUK) Gateway. Strengthening the Reporting of Observational Studies in Epidemiology (STROBE) guidance[44] and Reporting of studies Conducted using Observational Routinely-collected Data (RECORD) guidance[45] will be used to guide transparent reporting**.**

AUTHOR CONTRIBUTIONS

For peer review only

SJS, RW, CR and AS contributed to the conception of the study. All authors contributed to the study design. All authors contributed to drafting the protocol. All authors revised the manuscript for important intellectual content. All authors gave final approval of the version to be published.

PATIENT AND PUBLIC INVOLVEMENT

Parents and pregnant women have not been involved in design of this protocol. However, we will work in partnership with a patient and public involvement group set up for the EAVE II study regarding interpretation of results, presentation and dissemination of findings. We also have close links with Tommy's charity who will co-develop dissemination plans and help ensure that findings reach relevant stakeholders.

ACKNOWLEDGMENTS

The authors thank and acknowledge the wider EAVE II team for their support for this protocol.

FUNDING

EAVE II funded by the Medical Research Council (MR/R008345/1) with the support of BREATHE - The Health Data Research Hub for Respiratory Health [MC_PC_19004], which is funded through the UK Research and Innovation Industrial Strategy Challenge Fund and delivered through Health Data Research UK. Additional support has been provided through the Scottish Government DG Health and Social Care. COPS receive additional funding from Tommy's charity (1060508; SC039280). SJS is supported by Wellcome Trust (209560/Z/17/Z)

COMPETING INTERESTS

For peer review only - http://bmjopen.bmj.com/site/about/guidelines.xhtml

None declared.

DATA STATEMENT

Public Health Scotland and the Chief Medical Officer of Scotland are data controllers of the data used within the study. We will not be able to share data for the study as we are not the data controllers. All results put into the public domain will be subject to statistical disclosure control according to usual processes. Meta-data produced in this study will be made available to Health Data Research UK (HDRUK) Gateway. Applications to use the datasets included in the study can be made via https://www.informationgovernance.scot.nhs.uk/pbpphsc/

REFERENCES

1. Coronavirus (COVID-19) Infection in Pregnancy. 2020. Royal College of Obstetricians and Gynaecologists. Available from: https://www.rcog.org.uk/globalassets/documents/guidelines/2020-06-18-coronavirus-covid-19-infection-in-pregnancy.pdf. Accessed 18th July 2020.

2. Guidance on shielding and protecting people who are clinically extremely vulnerable from COVID-19. Public Health England. 2020. Available from: https://www.gov.uk/government/publications/guidance-on-shielding-and-protecting-extremely-vulnerable-persons-from-covid-19/guidance-on-shielding-and-protecting-extremely-vulnerable-persons-from-covid-19#who-is-clinically-extremely-vulnerable Accessed 14th July 2020

3. Di Mascio D, Khalil A, Saccone G, et al. Outcome of Coronavirus spectrum infections (SARS, MERS, COVID 1 -19) during pregnancy: a systematic review and meta-analysis. Am J Obstet Gynecol MFM 2020:100107.

For peer review only - http://bmjopen.bmj.com/site/about/guidelines.xhtml

4. If You Are Pregnant, Breastfeeding, or Caring for Young Children. Centers for Disease Control and Prevention. 2020. Available from: https://www.cdc.gov/coronavirus/2019-ncov/need-extra-precautions/pregnancy-breastfeeding.html?CDC_AA_refVal=https%3A%2F%2Fwww.cdc.gov%2Fcoronavirus%2F2019-ncov%2Fhcp%2Fpregnant-women-faq.html Accessed 14th July 2020

5. Huntley BJF, Huntley ES, Di Mascio D, et al. Rates of Maternal and Perinatal Mortality and Vertical Transmission in Pregnancies Complicated by Severe Acute Respiratory Syndrome Coronavirus 2 (SARS-Co-V-2) Infection: A Systematic Review. Obstet Gynecol 2020

6. Racicot K, Mor G. Risks associated with viral infections during pregnancy. J Clin Invest 2017;127(5):1591-99.

7. Coyne CB, Lazear HM. Zika virus - reigniting the TORCH. Nat Rev Microbiol 2016;14(11):707-15.

8. Zheng Q-L, Duan T, L-P. J. Single-cell RNA expression profiling of ACE2 and AXL in the human maternal–Fetal interface. Reprod Dev Med [Internet] 2020;2020 Mar 25 4(1):7.

9. Pique-Regi R, Romero R, Tarca AL, et al. Does the human placenta express the canonical cell entry mediators for SARS-CoV-2? bioRxiv 2020 May 18;2020.05.18.101485.

10. Hosier H, Farhadian S, Morotti R, et al. SARS-CoV-2 Infection of the Placenta. medRxiv 2020 May 12;2020.04.30.20083907.

11. Baud D, Greub G, Favre G, et al. Second-Trimester Miscarriage in a Pregnant Woman With SARS-CoV-2 Infection. JAMA 2020 doi: 10.1001/jama.2020.7233 [published Online First: 2020/05/01]

12. Mother-to-child transmission (MTCT). University of Birmingham COVID-19 in Pregnancy (PregCOV-19LSR). 2020. Available from:

For peer review only

https://www.birmingham.ac.uk/research/who-collaborating-centre/pregcov/about/mother-to-child-transmission.aspx Accessed 14th July 2020

13. Dong L, Tian J, He S, et al. Possible Vertical Transmission of SARS-CoV-2 From an Infected Mother to Her Newborn. JAMA 2020 doi: 10.1001/jama.2020.4621 [published Online First: 2020/03/28]

14. Zeng H, Xu C, Fan J, et al. Antibodies in Infants Born to Mothers With COVID-19 Pneumonia. JAMA 2020 doi: 10.1001/jama.2020.4861 [published Online First: 2020/03/28]

15. Multisystem inflammatory syndrome, Kawasaki disease and toxic shock syndrome. British Paediatric Surveillance Unit. 2020. Available from: https://www.rcpch.ac.uk/work-we-do/bpsu/study-multisystem-inflammatory-syndrome-kawasaki-disease-toxic-shock-syndrome Accessed 14th July 2020

16. Neonatal complications of coronavirus disease (COVID-19). British Paediatric Surveillance Unit. 2020. Available from: https://www.rcpch.ac.uk/work-we-do/bpsu/study-neonatal-complications-coronavirus-disease-covid-19 Accessed 14th July 2020

17. COVID-19 database (CO-CIN). International Severe Acute Respiratory and Emerging Infection Consortium (ISARIC). 2020. Available from: https://isaric.tghn.org/covid-19-clinical-research-resources Accessed 14th July 2020

18. Simpson CR, Robertson C, Vasileiou E, et al. Early Pandemic Evaluation and Enhanced Surveillance of COVID-19 (EAVE II): protocol for an observational study using linked Scottish national data. BMJ Open 2020;10(6):e039097.

19. Simpson CR, Beever D, Challen K, et al. The UK's pandemic influenza research portfolio: a model for future research on emerging infections. Lancet Infect Dis 2019;19(8):e295-e300.

20. Simpson CR, Thomas BD, Challen K, et al. The UK hibernated pandemic influenza research portfolio: triggered for COVID-19. Lancet Infect Dis 2020;20(7):767-69.

For peer review only - http://bmjopen.bmj.com/site/about/guidelines.xhtml

For peer review only

21. Da Silva Filipe A, Shepherd J, Williams T, et al. Genomic epidemiology of SARS-CoV-2 spread in Scotland highlights the role of European travel in COVID-19 emergence. medRxiv 2020;20124834.

22. Simpson CR, Ritchie LD, Robertson C, et al. Vaccine effectiveness in pandemic influenza - primary care reporting (VIPER): an observational study to assess the effectiveness of the pandemic influenza A (H1N1)v vaccine. Health Technol Assess 2010;14(34):313-46.

23. Public Health Scotland. Births in Scotland 2018-2019 2019. Available from: https://www.isdscotland.org/Health-Topics/Maternity-and-Births/Births/ Accessed 14th July 2020

24. Notification of Abortion Statistics (AAS). Public Health Scotland. 2020. Available from: https://www.ndc.scot.nhs.uk/National-Datasets/data.asp?SubID=64. Accessed 14th July 2020

25. Stillbirths and Infant Deaths. National Records of Scotland. 2020. Available from: https://www.nrscotland.gov.uk/statistics-and-data/statistics/statistics-by-theme/vital-events/deaths/deaths-background-information/stillbirths-and-infant-deaths Accessed 14th July 2020

26. Vital Events – Births. National Records of Scotland. 2020. Available from: https://www.nrscotland.gov.uk/statistics-and-data/statistics/statistics-by-theme/vital-events/births

27. Scottish Morbidity Record Type. Public Health Scotland. 2020. Available from: https://www.ndc.scot.nhs.uk/Data-Dictionary/SMR-Datasets/Episode-Management/SMR-Record-Type/ Accessed 14th July 2020

28. Scottish Birth Record. Public Health Scotland. 2020. Available from: https://www.isdscotland.org/Products-and-Services/Scottish-Birth-Record/ Accessed 14th July 2020

For peer review only

For peer review only - http://bmjopen.bmj.com/site/about/guidelines.xhtml

29. Scottish Intensive Care Society Audit Group Public Health Scotland. 2020. Available from: https://www.sicsag.scot.nhs.uk/index.html Accessed 14th July 2020

30. Communication of Surveillance in Scotland (ECOSS). Health Protection Scotland. 2020. Available from: https://www.hps.scot.nhs.uk/data/ Accessed 14th July 2020

31. Corona Virus 19 NHS Greater Glasgow and Clyde. 2020. Available from: https://www.nhsggc.org.uk/about-us/professional-support-sites/laboratory-medicine/laboratory-disciplines/microbiology-and-virology/west-of-scotland-specialist-virology-centre/# Accessed 14th July 2020

32. Community pathway for managing Covid-19 in Scotland- Delay phase. Scottish Government Heath and Social Care Directorates. 2020 Available from: https://www.sehd.scot.nhs.uk/publications/DC20200317Covid-19.pdf Accessed 14th July 2020

33. Urgent Care Data Mart (UCD) – Background Paper. Public Health Scotland. 2017. Available from: https://www.isdscotland.org/Health-Topics/Emergency-Care/Patient-Pathways/UrgentCareDataMartBackgroundPaper_20171002.pdf Accessed 14th July 2020

34. Vital Events – Deaths. National Records of Scotland. 2020. Available from: https://www.nrscotland.gov.uk/statistics-and-data/statistics/statistics-by-theme/vital-events/deaths Accessed 14th July 2020

35. Prescribing Information System (PIS). Public Health Scotland. 2020. Available from: https://www.ndc.scot.nhs.uk/National-Datasets/data.asp?SubID=9 Accessed 14th July 2020

36. ehealth. HEPMA [Available from: https://www.ehealth.scot/case-studies/hepma/.

37. Abortion Act 1967. UK Government. 1967. Available from: http://www.legislation.gov.uk/ukpga/1967/87/contents Accessed 14th July 2020

38. EUROCAT Guide 1.4: Instruction for the registration of congenital anomalies. In: EUROCAT Central Registry, ed. University of Ulster, 2013.

For peer review only

39. Wright CM, Booth IW, Buckler JM, et al. Growth reference charts for use in the United Kingdom. Arch Dis Child 2002;86(1):11-4.

40. Scottish Index of Multiple Deprivation. Scottish Government. 2020. Available from: https://www.gov.scot/collections/scottish-index-of-multiple-deprivation-2020/ Accessed 14th July 2020

41. Scottish urban/rural 8 fold classification. Scottish Government. 2020. Available from: https://www2.gov.scot/Topics/Statistics/About/Methodology/UrbanRuralClassification Accessed 14th July 2020

42. COVID-19 - search criteria for highest risk patients for shielding. Health Protection Scotland. 2020. Available from: https://www.hps.scot.nhs.uk/web-resources-container/covid-19-search-criteria-for-highest-risk-patients-for-shielding/ Accessed 14th July 2020

43. WHO. Clinical Guidelines on the Identification, Evaluation, and Treatment of Overweight and Obesity in Adults--The Evidence Report. National Institutes of Health Obes Res 6 Suppl 2:51S-209S 1998

44. von Elm E, Altman DG, Egger M, et al. Strengthening the Reporting of Observational Studies in Epidemiology (STROBE) statement: guidelines for reporting observational studies. BMJ 2007;335(7624):806-8.

45. Benchimol EI, Smeeth L, Guttmann A, et al. The REporting of studies Conducted using Observational Routinely-collected health Data (RECORD) statement. PLoS Med 2015;12(10):e1001885.

46. COVID-19 in Pregnancy. UKOSS University of Oxford.2020. Available from: https://www.npeu.ox.ac.uk/ukoss/current-surveillance/covid-19-in-pregnancy Accessed 14th July 2020

47. Pregnancy And Neonatal outcomes for women with COVID-19 (PAN-COVID). Imperial College London. 2020. Available from: https://pan-covid.org Accessed 14th July 2020

For peer review only - http://bmjopen.bmj.com/site/about/guidelines.xhtml

48. PERICOVID. Public Health England. 2020. Available from: https://www.pericovid.com Accessed 14th July 2020

49. Coronavirus (COVID-19) data. Public Health Scotland. 2020. Available from: https://publichealthscotland.scot/our-areas-of-work/sharing-our-data-and-intelligence/coronavirus-covid-19-data/ Accessed 14th July 2020

For peer review only

TABLES

Table 1: UK surveillance studies on COVID-19 in pregnant women and their babies

Name of study	Institution	Inclusion	Reporting by	Consent required	Likely coverage in Scotland
COVID-19 in Pregnancy [46]	UK Obstetric Surveillance System study (UKOSS)	Any women admitted to hospital in the UK with confirmed COVID-19 at any stage of pregnancy	Front-line clinicians	No	High
Pregnancy And Neonatal outcomes for women with COVID-19 (PAN-COVID) [47]	National Institute of Healthcare Research (NIHR) Imperial Biomedical Research Centre	Women who have suspected or confirmed COVID-19 at any stage during pregnancy and their babies	Front-line clinicians	Yes	Unknown as yet
Clinical Characterisation Protocol Tier 0	The International Severe Acute	Any patient admitted participating	Reporting is by research nurses	No	Low but may increase

For peer review only

study (CO-CIN) [17]	Respiratory and emerging Infection Consortium (IASRIC)	hospitals in the UK with confirmed COVID-19			
Neonatal complications of coronavirus disease (COVID-19) [17]	British Paediatric Surveillance Unit (BPSU)	All babies born to mothers with COVID-19 who are admitted to neonatal care (whether the baby has COVID-19 or not) and all babies with confirmed COVID-19 in the neonatal period.	Front-line clinicians.	No	High
Multisystem inflammatory syndrome, Kawasaki disease and toxic	British Paediatric Surveillance Unit (BPSU)	All children less than 16 years old (including neonates) with	Front-line clinicians	No	High

For peer review only

shock syndrome[15]		multisystem inflammatory syndrome due to SARS-CoV-2 infection or otherwise unexplained.			
Understanding COVID-19 infection in women and their babies (periCOVID)[48]	Public Health England and St George's University London	Any pregnant woman with confirmed COVID-19 infection from 24 weeks gestation in England	Clinicians/research midwives and nurses	Yes	None

For peer review only

Table 2: Estimated number of confirmed COVID-19 cases March to May 2020 in pregnant women in Scotland

	Total number of individuals testing positive (PCR) for SARS-CoV-2 (NHS labs only)	Women aged 15-44 years testing positive (PCR) for SARS-CoV-2 (NHS labs only)	Estimated number of pregnant women testing positive (PCR) for SARS-CoV-2 (NHS labs only)**
March 2020	≈2000	≈333*	≈17
April 2020	≈9000	≈1500*	≈75
May 2020	≈4000	≈667*	≈33
Total	≈15000	≈2500	≈125

* Assuming the distribution over time for this age/sex group is the same as for all tests, as age/sex breakdown only available from published information[49] for the total

** Assuming that around 5% of the female population aged 15-44 is pregnant at any one time, and that incidence of COVID-19 is the same in pregnant and non-pregnant women

For peer review only

FIGURE LEGENDS

Figure 1: Overview of data linkage for the Covid-19 in Pregnancy in Scotland (COPS) study

For peer review only

For peer review only - http://bmjopen.bmj.com/site/about/guidelines.xhtml

Figure 1: Overview of data linkage for the Covid-19 in Pregnancy in Scotland (COPS) study

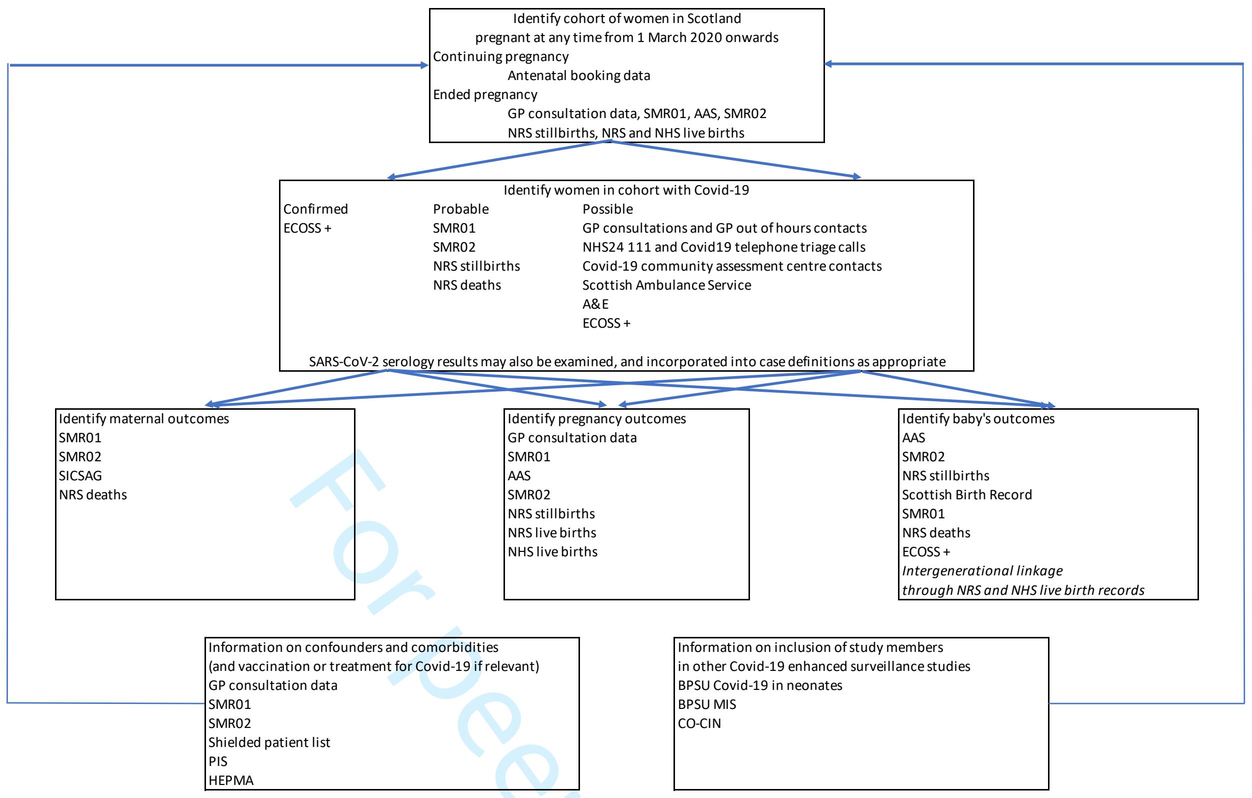

For peer review only

Supplementary Material: Additional information on identifying pregnant women, and associated start and end of pregnancy dates, from national data sources

National data sources identifying end of pregnancy events

There are three possible outcomes for any pregnancy

Pregnancy outcome	Comments
Spontaneous loss	'Miscarriage' at <24w (here taken to include ectopic pregnancies, although mechanisms underlying spontaneous miscarriage and ectopic pregnancy differ)<br>(Sometime 'late fetal loss' at 20-23w as a subset of miscarriages)<br>'Stillbirth' at ≥24w
Termination of pregnancy	Legal at <24w under Grounds C and D of the Abortion Act 1967<br>Legal at any gestation under Grounds A, B, E, F, G
Live birth	No lower gestational limit although in practice around 22w would be considered the lower limit at which live born babies may survive

For peer review only

Various national records may be returned following these end of pregnancy events, as summarised below

National record	Description	Pregnancy outcomes identified	Coding to identify relevant records
Identifying spontaneous pregnancy losses			
SMR01	Record of day case or inpatient admission to any general unit (excluding neonatal, maternity, and mental health care), including admissions under gynaecology specialty	Will identify early (first trimester) spontaneous losses managed in hospital in most Board areas	ICD10:<br>O00 (ectopic pregnancy)<br>O01 (hydatidiform mole)<br>O02 (missed miscarriage)<br>O03, O05, O06 (spontaneous miscarriage), all .5-.9
OR<br>SMR02	Record of day case or inpatient admission to a maternity unit, including admissions under obstetrics or midwifery specialties	Will identify early (first trimester) spontaneous losses managed in hospital in some Board areas<br>Will identify later (second and third trimester) spontaneous losses managed in hospital in all areas	Miscarriages<br>Condition on discharge=2 (aborted)<br>Type of abortion=1, 2, 3, 6, 8, 9 (spontaneous)<br><br>Stillbirths<br>Condition on discharge=3 (delivered)<br>Outcome of pregnancy=2 (stillbirth)
AND/OR<br>NRS stillbirths	Record of statutory registration of a stillbirth (baby born at ≥24w showing no signs of life)	Will identify spontaneous stillbirths	ICD10:<br>P96.4 not recorded
Identifying terminations of pregnancy			
AAS	Record of statutory notification of a termination of pregnancy	Should identify all terminations of pregnancy but known under-notification of later ToPs done for fetal anomaly from some maternity units	

AND/OR<br>SMR02	As above	Will identify later ToPs done for fetal anomaly in maternity units	Condition on discharge=2 (aborted)<br>Type of abortion=4 (ToP)
AND/OR<br>NRS stillbirths	As above	Will identify the small number of stillbirths following a termination of pregnancy	ICD10:<br>P96.4 recorded in any position
Identifying live births			
SMR02	As above	Will identify live births occurring in hospital<br>SMR02 returns were enabled to cover home (as well as in hospital) births from Apr 2019, and coverage of home births should have been mandatory from Oct 2019, however technical difficulties mean that home births are still (as at July 2020) not recorded on SMR02 in most Boards	Condition on discharge=3 (delivered)<br>Outcome of pregnancy=1, 3, 4, 5 (live birth)
AND/OR<br>NRS live births	Record of statutory registration of a live birth (live born baby at any gestation)	Usually identifies all live births however statutory registration of live births was suspended from 23 March to 28 June 2020 inclusive when registrar offices closed<br>The only babies being registered during that period were those that subsequently die: this was done remotely along with the death registration to avoid parents having to register the birth in person later<br>A catch up programme of live birth registrations started on 29 June 2020	

For peer review only

AND/OR<br><br>NHS live birth notifications	Notification of live births from NHS Board maternity units to child health administration departments<br><br>This notification allows a record to be created for the child on the national child health information system: this in turn ensures the child is called for immunisations and child health reviews	As NRS live birth registration was suspended in March – June 2020 due to COVID-19 (see above), PHS has recently developed a new data extraction from the national child health information system of birth notification data<br><br>This will identify all live births known to NHS maternity services from Aug 2019 onwards<br><br>A small number of babies who die very soon after birth (before that day's notification data has been sent) will not be included as these babies do not need to be notified for ongoing care, however they will be covered by NRS registration as noted above	

It is possible that the same woman/pregnancy may have multiple records giving conflicting information on the outcome of the pregnancy.

In general, if any record indicates a termination of pregnancy, this should be taken as the outcome.

If an NRS stillbirth record is available for a baby but the corresponding SMR02 record indicates the baby was live born, this should be taken as a stillbirth.

For peer review only

The relevant gestation and date of event information in the various records, and how to deal with missing gestation information, is summarised below

National record	Gestation information available	Date of event information available	Dealing with missing gestation information (due to not recorded on that record, missing, or recorded but unfeasible)
SMR01	None	Date of admission<br>Date of discharge	Assume 12 weeks gestation at date of admission
SMR02	Gestation in completed weeks at end of pregnancy available on records where Condition on discharge=2 or 3 (aborted or delivered)	Date of admission<br>Date of discharge<br>Date of delivery on records where Condition on discharge=3 (delivered)	Miscarriage records with missing gestation, assume 12 weeks gestation at date of admission<br>ToP records with missing gestation (and not available from AAS), assume 16 weeks gestation at date of admission<br>Stillbirth delivery records with missing gestation (and not available from NRS), assume 32 weeks gestation at date of delivery<br>Live birth delivery records with missing gestation, assume 40 weeks gestation at date of delivery
NRS stillbirths	Gestation in completed weeks at date of stillbirth available	Date of stillbirth	Assume 32 weeks gestation at date of delivery (if not available from SMR02)
AAS	Gestation in completed weeks at date of termination available	Date of termination (date of administration of antiprogesterone for medical ToPs)	Assume 10 weeks gestation at date of termination (if not available from SMR02)
NRS live births	None	Date of birth	Assume 40 weeks gestation at date of birth (if not available from SMR02)

For peer review only

NHS live birth notifications	Gestation in completed weeks at date of birth available<br>(Although note this data has not been used before by PHS so will require checking before use)	Date of birth	Assume 40 weeks gestation at date of birth (if not available from SMR02)

For peer review only

The time lag inherent in the different data returns is summarised below

National record	Time lag inherent in data source
SMR01	Records should be returned to PHS within 6 weeks of patient's discharge (in practice sometimes longer)<br>Monthly batches (all records received to that point) are then uploaded to the analysis platform (SMRA) around the middle of each month<br>Records are CHI seeded as they are uploaded to the analysis platform (and CHI seeding is refreshed monthly thereafter to pick up any previously unseeded records)<br>CHI seeding usually complete on first attempt<br>So: records relating to discharges in Jan XX should be available for linkage and analysis within PHS in mid Apr XX (2.5 month lag)
SMR02	Records should be returned to PHS within 6 weeks of patient's discharge (in practice sometimes longer)<br>Monthly batches (all records received to that point) are then uploaded to the analysis platform around the middle of each month<br>Records are CHI seeded as they are uploaded to the analysis platform (and CHI seeding is refreshed monthly thereafter to pick up any previously unseeded records)<br>Maternal CHI seeding usually complete on first attempt<br>Baby CHI seeding usually complete on second attempt<br>So: as linkage of SMR02 records is generally through maternal CHI, records relating to discharges in Jan XX should be available for linkage and analysis within PHS in mid Apr XX (2.5 month lag)

For peer review only

NRS stillbirths	Registration required within 21 days of birth<br>Data transferred by NRS to PHS weekly<br>Monthly batches (stillbirths registered up to the end of the previous month) are then uploaded to the analysis platform around the middle of each month<br>In parallel, records are sent to NHSCR monthly for seeding of maternal CHI<br>As seeded records are returned from NHSCR, the CHIs are added to the records on the analysis platform<br>So: records relating to stillbirths occurring in Jan XX should be available for linkage and analysis within PHS in mid May XX (3.5 month lag)<br>(Note: almost all stillbirths will have an SMR02 record so can be identified and linked with 2.5 month lag)
AAS	Notification to CMO required within 7 days of termination<br>Records forwarded to PHS and entered into AAS system (includes automated CHI seeding) within 6 weeks of date of termination<br>So: records relating to terminations occurring in Jan XX should be available for linkage and analysis within PHS in mid Mar XX (1.5 month lag)

For peer review only

NRS live births	Registration required within 21 days of birth<br>Data transferred by NRS to PHS weekly<br>Monthly batches (live births registered up to the end of the previous month) are then uploaded to the analysis platform around the middle of each month<br>Records are seeded with baby CHI as they are uploaded to the analysis platform (and CHI seeding is refreshed monthly thereafter to pick up any previously unseeded records)<br>Baby CHI seeding usually complete on second attempt<br>In parallel, monthly batches are seeded with the mother's CHI by bespoke linkage to SMR02 after a 6 month lag (i.e. records for births in Jan XX and matched against SMR02 in Jul XX)<br>Records with no maternal CHI found are then matched against the full CHI database<br>Residual records with still no maternal CHI are then sent to NHSCR in monthly batches<br>So: as linkage of NRS live birth records generally requires both maternal and baby CHI (to allow intergenerational linkage), records relating to births in Jan XX should be available for linkage and analysis within PHS in mid Oct XX (8.5 month lag)<br>(Note: all live births from Aug 2019 onwards will have a birth notification record available so can be identified and linked with a 1 month lag)
NHS live birth notifications	Live births are notified to the NHS Board child health admin department within 1 working day of date of birth and are keyed into the national child health info system promptly (same or subsequent day)<br>PHS extracts notification data (including baby's CHI) from the national child health info system weekly<br>Maternal CHI is then seeded onto the data extracts weekly<br>So: records relating to births in Jan XX should be available for linkage and analysis within PHS in Feb XX (1 month lag)

For peer review only

National data sources identifying continuing pregnancies as early as possible

As part of the response to COVID-19, PHS has established a new national data return providing information on women booking for antenatal care. This will allow us to identify pregnant women before the end of their pregnancy, and hence monitor SARS-CoV-2 infections occurring in pregnant women in closer to real time. Further information on this data source is provided below.

Data items being requested in the new data feed include

- Maternal CHI
- Mother's Forename, Surname, Date of Birth, and Postcode in case CHI is missing and needs to be appended
- Date of Booking
- Gestation at booking
- Date of Last Menstrual Period (in case gestation is missing)

PHS has asked NHS Boards to provide an initial submission of historic data on all women booking from 1 April 2019, then subsequent weekly updates. The weekly updates will give information on women who have booked in the most recent week, and also update any records relating to the previous 2 weeks if those have changed since the previous submission. The current assumption is that this data will be submitted with maternal CHI complete, hence additional lag for CHI seeding will not be required but this is being kept under review.

This dataset will identify all women booking for NHS antenatal care. The method of providing booking services has changed in many areas due to COVID-19, with many Boards now providing the initial booking appointment remotely, with the woman subsequently attending in person for her initial ultrasound scan and blood tests[1]. To ensure that the dataset allows us to identify pregnant women as early as possible in their maternity care journey, the 'booking' event that is captured in the above dataset has therefore been defined as '*the date on which maternity services had the first planned/structured contact with a pregnant woman to assess her history and needs so that local maternity services can provide further care such as an early pregnancy scan and antenatal screening tests*', i.e. the initial remote contact.

[1] https://tec.scot/clinical-specialty-guidance/

For peer review only

Available national data shows that, pre-COVID-19, at least 90% of pregnant woman attended their booking appointment by 12^{+6} weeks gestation[2]. There is currently no evidence that gestation at initial booking has increased due to COVID-19. If gestation and LMP are both missing on antenatal booking records, we will therefore assume the woman was at 12 weeks gestation at the date of booking.

[2] https://www.isdscotland.org/Health-Topics/Maternity-and-Births/Publications/

For peer review only

Defining start and end date of pregnancies

For pregnancies that have ended

Pregnancy end dates will be taken from end of pregnancy records as noted above

Pregnancy start date (date of conception) will be imputed from the pregnancy end date and the gestation at pregnancy end – 2 weeks

For continuing pregnancies

Pregnancy start date (date of conception) will be imputed from the date of antenatal booking and the gestation at booking – 2 weeks, or from the date of last menstrual period + 2 weeks if gestation is missing and LMP is provided

Time lags inherent in data sources identifying COVID-19 status and relevant outcomes

In general, the time lags inherent in data sources identifying COVID-19 status and relevant outcomes are less than (or at least no more than) those inherent in the various data sources required to identify pregnancy status.

The only additional lag that needs to be considered is that seen in Scottish Birth Record (SBR) records. SBR records are not returned to PHS as such. Rather, PHS takes a monthly download of data held on the system for analysis purposes. In most NHS Boards, the SBR system is used to generate a CHI number for a baby shortly after birth. Skeleton records with minimal demographic data are therefore available for all babies in a timely manner. For babies admitted to neonatal care, clinical coding staff within NHS Board admin departments are responsible for completing additional variables within a baby's SBR record following their discharge. There is no national standard for when this should be done and in practice the lag between discharge and a completed record being available varies between Boards. Some Boards achieve broadly complete records within 3 months whereas others take considerably longer. Currently (June 2020) NHS Borders and NHS Dumfries & Galloway have not coded any SBR records (or provided comparable data directly to PHS) since June 2017 and April 2018 respectively. SBR data is therefore unlikely to provide a complete picture of neonatal admissions within the timeframes set out for this analysis (i.e. for babies born in March 2020, the data available to PHS on SBR by July 2020 will only provide a partial picture of admissions to neonatal care). PHS may explore getting a new national feed from NHS Boards of more real time data on neonatal admissions to mitigate this problem if feasible.

For peer review only

BMJ Open

COVID-19 in Pregnancy in Scotland (COPS): protocol for an observational study using linked Scottish national data

Journal:	*BMJ Open*
Manuscript ID	bmjopen-2020-042813.R1
Article Type:	Protocol
Date Submitted by the Author:	24-Sep-2020
Complete List of Authors:	Stock, Sarah; The University of Edinburgh, Usher Institute; The University of Edinburgh MRC Centre for Reproductive Health, Tommy's Centre for Maternal and Fetal Health<br>McAllister, David; University of Glasgow; Public Health Scotland, David McAllister<br>Vasileiou, Eleftheria; The University of Edinburgh, Usher Institute;<br>Simpson, Colin; Victoria University of Wellington,<br>Stagg, Helen R.; University of Edinburgh, Usher Institute<br>Agrawal, Utkarsh; University of St Andrews, School of Medicine<br>McCowan, Colin ; University of St. Andrews<br>Hopkins, Leanne; Public Health Scotland<br>Donaghy, Jack; Public Health Scotland<br>Ritchie, Lewis; Aberdeen University, General Practice and Primary Care<br>Robertson, Chris; University of Strathclyde, Department of Mathematics and Statistics<br>Sheikh, Aziz; University of Edinburgh, Division of Community Health Sciences<br>Wood, Rachael; NHS National Services Scotland, Information Services Division; University of Edinburgh, Child Life and Health
**Primary Subject Heading**:	Epidemiology
Secondary Subject Heading:	Obstetrics and gynaecology, Infectious diseases
Keywords:	COVID-19, Epidemiology < INFECTIOUS DISEASES, OBSTETRICS, NEONATOLOGY, PERINATOLOGY

SCHOLARONE™
Manuscripts

I, the Submitting Author has the right to grant and does grant on behalf of all authors of the Work (as defined in the below author licence), an exclusive licence and/or a non-exclusive licence for contributions from authors who are: i) UK Crown employees; ii) where BMJ has agreed a CC-BY licence shall apply, and/or iii) in accordance with the terms applicable for US Federal Government officers or employees acting as part of their official duties; on a worldwide, perpetual, irrevocable, royalty-free basis to BMJ Publishing Group Ltd ("BMJ") its licensees and where the relevant Journal is co-owned by BMJ to the co-owners of the Journal, to publish the Work in this journal and any other BMJ products and to exploit all rights, as set out in our licence.

The Submitting Author accepts and understands that any supply made under these terms is made by BMJ to the Submitting Author unless you are acting as an employee on behalf of your employer or a postgraduate student of an affiliated institution which is paying any applicable article publishing charge ("APC") for Open Access articles. Where the Submitting Author wishes to make the Work available on an Open Access basis (and intends to pay the relevant APC), the terms of reuse of such Open Access shall be governed by a Creative Commons licence – details of these licences and which Creative Commons licence will apply to this Work are set out in our licence referred to above.

Other than as permitted in any relevant BMJ Author's Self Archiving Policies, I confirm this Work has not been accepted for publication elsewhere, is not being considered for publication elsewhere and does not duplicate material already published. I confirm all authors consent to publication of this Work and authorise the granting of this licence.

For peer review only

COVID-19 in Pregnancy in Scotland (COPS): protocol for an observational study using linked Scottish national data

Sarah J Stock, Usher Institute, University of Edinburgh NINE Edinburgh BioQuarter, 9 Little France Road, Edinburgh EH16 4UX UK sarah.stock@ed.ac.uk +44 (0)7894629934 (corresponding author) ORCID ID 0000-0003-4308-856X

David McAllister, Public Health Scotland, UK. University of Glasgow, Glasgow, UK, ORCID ID 0000-0003-3550-1764

Eleftheria Vasileiou, Usher Institute, University of Edinburgh, Edinburgh, UK ORCID ID 0000-0001-6850-7578

Colin R Simpson, School of Health, Wellington Faculty of Health, Victoria University of Wellington, Wellington, New Zealand; Usher Institute, University of Edinburgh, Edinburgh, UK ORCID ID 0000-0002-5194-8083

Helen R Stagg, Usher Institute, University of Edinburgh, Edinburgh, UK ORCID ID 0000-0003-4022-3447

Utkarsh Agrawal, School of Medicine, University of St Andrews, St Andrews, UK ORCID ID 0000-0001-5181-6120

Colin McCowan, School of Medicine, University of St Andrews, St Andrews, UK ORCID ID 0000-0002-9466-833X

Leanne Hopkins, Public Health Scotland, UK ORCID ID 0000-0002-7487-4363

Jack Donaghy, Public Health Scotland, UK ORCID ID 0000-0002-6137-1601

For peer review only

For peer review only - http://bmjopen.bmj.com/site/about/guidelines.xhtml

Lewis Ritchie, Institute of Applied Health Sciences, University of Aberdeen, UK ORCID ID 0000-0002-9380-7641

Chris Robertson, Public Health Scotland, UK. Department of Mathematics and Statistics, University of Strathclyde, Glasgow, UK ORCID ID 0000-0001-6848-5241

Aziz Sheikh, Usher Institute, University of Edinburgh, Edinburgh, UK ORCID ID 0000-0001-7022-3056

Dr Rachael Wood, Public Health Scotland and University of Edinburgh, UK ORCID ID 0000-0003-4453-623X

Key Words: COVID-19, Pregnancy, Maternal, Neonatal, Perinatal, Coronavirus

Dates of study: 25th August 2020 to 30th September 2021

Word Count: 5166

For peer review only

ABSTRACT

Introduction

The effects of SARS-CoV-2 in pregnancy not fully delineated. We will describe the incidence of COVID-19 in pregnancy at population level in Scotland, in a prospective cohort study using linked data. We will determine associations between COVID-19 and adverse pregnancy, neonatal and maternal outcomes; and the proportion of confirmed cases of SARS-CoV-2 infection in neonates associated with maternal COVID-19.

Methods and analysis

Prospective cohort study using national linked datasets. We will include all women in Scotland, UK, who were pregnant on, or became pregnant after, 1st March 2020 (the date of the first confirmed case of SARS-CoV-2 infection in Scotland), and all births in Scotland from 1st March 2020 onwards. Individual-level data will be extracted from datasets containing details of all livebirths, stillbirth, terminations of pregnancy, and miscarriages and ectopic pregnancies treated in hospital or attending general practice. Records will be linked within the Early Pandemic Evaluation and Enhanced Surveillance of COVID-19 (EAVE II) platform, which includes primary care records, virology and serology results, and details of COVID-19 Community Hubs and Assessment Centre contacts and deaths. We will perform analyses using definitions for confirmed, probable and possible COVID-19, and report serology results (where available). Outcomes will include congenital anomaly, miscarriage, stillbirth, termination of pregnancy, preterm birth, neonatal infection, severe maternal disease and maternal deaths. We will perform descriptive analyses and appropriate modelling, adjusting for demographic and pregnancy characteristics, and the presence of co-morbidities. The cohort will provide a platform for future studies of the effectiveness and safety of

For peer review only

therapeutic interventions and immunisations for COVID-19, and their effects on childhood and developmental outcomes.

Ethics and dissemination

COPS is a sub-study of EAVE II, which has approval from the National Research Ethics Service Committee. Findings will be reported to Scottish Government, Public Health Scotland and published in peer reviewed journals.

For peer review only

For peer review only - http://bmjopen.bmj.com/site/about/guidelines.xhtml

ARTICLE SUMMARY

Strengths and limitations of this study

- We will interrogate Scottish national data at the population level to provide information on the incidence of, and outcomes following, COVID-19 outcomes in pregnant women.
- We are expanding an existing national pandemic reporting platform (EAVE II) to include assessment of all pregnancy outcomes. EAVE II uses de-identified individual patient-level data for almost the entire population of Scotland from general practices, hospitals, death registry, virology (Reverse Transcriptase Polymerase Chain Reaction; RT-PCR) and serology tests to investigate the epidemiology of COVID-19.
- This is an observational study and residual confounding is a potential concern.

For peer review only

For peer review only - http://bmjopen.bmj.com/site/about/guidelines.xhtml

INTRODUCTION

The effects of novel Severe Acute Respiratory Syndrome Coronavirus -2 (SARS-CoV-2) in pregnancy are yet to be fully delineated.[1] Pregnant women are at greater risk of complications and severe disease from infection with other coronaviruses including SARS and Middle Eastern Respiratory Syndrome (MERS).[2 3] Pregnant women were thus identified as a potential vulnerable group in some countries and advised to take additional precautions as the Coronavirus Disease 2019 (COVID-19) pandemic unfolded.[2 3 4]

To inform public health policy, it is crucial to determine the effects of SARS-CoV-2 infection on maternal, pregnancy, and neonatal health. SARS-CoV-2 transmission from mother to baby (antenatally or intrapartum) appears to be possible,[5] but the proportion of pregnancies affected and the clinical significance is uncertain. Potential effects of the virus on miscarriage, congenital anomalies, fetal growth, timing of delivery, and stillbirth are unknown. We know from other viral infections in pregnancy that infections with mild maternal symptomatology can have substantial impacts on the developing fetus (e.g. Cytomegalovirus, Parvovirus, Zika virus),[6] although mechanisms of placental transmission of virus vary.[7] The canonical receptors for SARS-CoV-2 (the angiotensin- converting enzyme 2 [ACE2] receptor and the serine protease TMPRSS2) are not co-expressed in the placenta, making placental infection unlikely.[8 9] Nevertheless, case reports have shown evidence suggestive of viral infection of the placenta, in association with pregnancy complications such as pre-eclampsia and abruption[10] and second trimester miscarriage.[11] Reports that include neonatal test results for SARS-CoV-2 show positive cases only in a minority of babies, with significant respiratory disease being rare in neonates.[12] However, some babies born to mothers who had COVID-19 have increased concentrations of both immunoglobulin IgM and IgG for SARS-CoV-2.[13 14] As IgM cannot cross the placenta, neonatal circulating

For peer review only

SARS-CoV-2 IgM indicates vertical transmission of virus, although all the infants in reports so far have been asymptomatic and tested negative for SARS-CoV-2 viral RNA at birth.[13 14] There are also plausible links between SARS-CoV-2 and pregnancy complications such as preterm birth, which may be mediated either as a manifestation of COVID-19 disease itself; or indirectly through increased stress due to the pandemic and containment measures, or through altered physician threshold for iatrogenic preterm delivery in women with infection.[1]

Understanding the effects of COVD-19 at different stages in pregnancy and perinatally will help inform policy on shielding strategies, and advice to pregnant women and those considering pregnancy. It is also essential to inform immunisation strategies when vaccines are available. For example, immunisation in early pregnancy may help protect against maternal infection during pregnancy and reduce complications; but immunisation in later pregnancy may be preferential to provide passive immunisation to babies if neonatal infection is the predominant concern.

There are a number of surveillance studies gathering data on pregnant women with COVID-19 currently underway in the UK, summarised in Table 1.

Collectively, these surveillance studies can provide detailed characterisation of selected groups of pregnant women (and neonates) affected by COVID-19. The study outlined in this protocol will complement these existing studies by providing population-based information (for the whole of Scotland) on the risks of, and outcomes following, COVID-19 at any stage of pregnancy for women in the community and/or admitted to hospital.

The primary objectives of the COVID-19 in Pregnancy in Scotland (COPS) study are to:

a) describe the incidence of SARS-CoV-2 infection and COVID-19, in the pregnant population;

For peer review only

b) determine associations between COVID-19 and adverse maternal, pregnancy, and neonatal outcomes;

c) determine the proportion of neonates with confirmed SARS-CoV-2 infection that are associated with COVID-19 in the baby's mother

Secondary objectives are to:

a) assess the proportion of COVID-19 cases in pregnant women and neonates who are included in relevant other enhanced surveillance studies (e.g. BPSU, CO-CIN);[15-17]

b) provide a platform to assess the safety and effectiveness of any new or existing prophylactic or therapeutic interventions (e.g. new or repurposed therapies, vaccines, antimicrobials) in pregnant women and their babies;

c) enable evaluation of the longer-term sequelae of maternal SARS-CoV-2, and therapeutic interventions to mitigate SARS-CoV-2 in pregnancy and in children.

This COPS study is a sub-study of the EAVE II study (Early Pandemic Evaluation and Enhanced Surveillance of COVID-19 observational study using linked Scottish national data).[18-20] EAVE II is a national, real-time, data platform to identify the population groups most at risk from SARS-CoV-2 infection and COVID-19 disease and mortality, linking Scottish General Practice (GP) records (5.4 million registered patients) with secondary care and laboratory datasets.[18] Pregnancy will be assessed as one of these at-risk groups. Within this COPS study protocol, we specify in detail the national datasets that will be incorporated within the EAVE II platform to enable pregnant women (and associated pregnancy start and end dates) to be reliably identified. We also specify in detail the maternal, pregnancy and neonatal outcomes following maternal COVID-19 that will be examined.

For peer review only - http://bmjopen.bmj.com/site/about/guidelines.xhtml

METHODS

Patient and public involvement

Parents and pregnant women have not been involved in design of this protocol. However, we will work in partnership with a patient and public involvement group set up for the EAVE II study regarding interpretation of results, presentation and dissemination of findings. We also have close links with Tommy's charity who will co-develop dissemination plans and help ensure that findings reach relevant stakeholders.

Study design and population

This is a prospective cohort study using national maternity, community, hospital and laboratory linked datasets, in Scotland, UK. We will include all women in Scotland who were pregnant on, or became pregnant after 1st March 2020 (the date of the first confirmed case of SARS-CoV-2 infection in Scotland[21]), and all live born babies born in Scotland from 1st March 2020 onwards. The end date for the study will be determined by the future development, and in particular suppression of, the pandemic in Scotland.

Women in the cohort with the earliest dates of conception will only have been at risk of COVID-19 at the very end of their pregnancy. Women with more recent dates of conception will have been at risk for longer, up to women with date of conception from 1 March 2020 onwards who will be at risk from conception onwards (until viral transmission is completely suppressed).

We aim to use a dynamic cohort of the entire pregnant population; therefore, selection bias is not anticipated and the dataset will be fully generalisable to Scotland (with extensive generalisability to other high-income nations).

For peer review only

Databases

Individual-level data will be extracted from the datasets listed below. Records relating to the same individual will be linked deterministically using the Community Health Index (CHI) number.[22] The CHI number is a unique identifier provided by National Health Service (NHS) Scotland for each resident registered with a general practice. Public Health Scotland routinely adds both maternal and baby CHI numbers to live birth registration records: these 'spine' records will be used to facilitate intergenerational linkage of records relating to mothers and their babies.

An overall schema for the planned linkage is provided in Figure 1. Unless otherwise specified, included datasets are held by Public Health Scotland (PHS) and PHS is the data controller.

Datasets to identify pregnant women in the general population and associated pregnancy start and end dates, and pregnancy and neonatal outcomes

i) Antenatal booking records: This is a new national data return developed as part of the response to the COVID-19 pandemic providing information on all women booking for antenatal care with NHS maternity services throughout Scotland. It will be used for identification of women with ongoing pregnancies in near real-time. All other records identify end of pregnancy events, thus there is a time-lag before records are generated. More than 99% of births in Scotland book for antenatal care with NHS maternity services.[23]

ii) Abortion Act Scotland [AAS] records: These are statutory notifications of termination of pregnancy, and will be used to identify all terminations of pregnancy, and including terminations of pregnancy indicated by congenital anomaly.[24]

For peer review only

For peer review only - http://bmjopen.bmj.com/site/about/guidelines.xhtml

iii) National Records of Scotland (NRS) statutory stillbirth registrations: Scottish legislation requires all stillbirths at 24 weeks gestation or more to be registered with NRS.[25]

iv) NRS statutory live birth registrations: Scottish legislation requires all live births at any gestation to be registered with NRS.[26]

v) NHS Scottish health board live births: A new national data return developed as part of the response to the COVID-19 pandemic (specifically to mitigate unavailability of NRS statutory live birth registration records when registration processes were suspended) providing information on all live births notified by maternity services to NHS Board child health administrative departments.

vi) GP data: Data from all patients registered in general practices are included in the EAVE II platform.[18] In COPS, these records will be used for identification of women with early miscarriage or ectopic pregnancy not managed in hospitals, and potential confounding co-morbidities.

vii) Scottish Morbidity Record (SMR) 01: The SMR01 database includes all general day case and in-patient admissions in Scotland.[27] Admissions to neonatal, maternity, and mental health care are excluded from SMR01 as they are covered by other specialist datasets. SMR01 records are included in the EAVE II platform, and in COPS will be used for identification of women with early miscarriage or ectopic pregnancy managed in hospitals, and potential confounding co-morbidities.

viii) SMR 02: The SMR02 database includes all day case and in-patient admissions to maternity specialties in Scotland.[27] It will be used for identification of later miscarriage, stillbirth, and live births managed in maternity units ($\geq$98% of births in Scotland) and some home births ($\leq$2% of births in Scotland).[23]

For peer review only - http://bmjopen.bmj.com/site/about/guidelines.xhtml

ix) Scottish Birth Record (SBR): The SBR records basic demographic data on all births in Scotland, and additional clinical information and diagnostic and operational procedure codes on babies admitted to neonatal care.[28] It will be used to identify neonates admitted to neonatal care.

x) Scottish Intensive Care Society Audit Group (SICSAG) records: This is a national database of patients admitted to adult general critical care units in Scotland detailing information on the management of critically ill or injured patients. All general Intensive Care Units and combined ICU/High Dependency Units (HDU) collect data and more than 90% of general High Dependency Units and a number of specialist ICU and HDUs also provide records.[29] In COPS these will be used to identify women admitted to critical care with COVID-19.

Datasets to identify women with confirmed SARS-CoV-2 infection or COVID-19

i) Electronic Communication of Surveillance in Scotland (ECOSS)[30] and other viral RT-PCR and serology results held separately by PHS are included on the EAVE II platform.[18] In COPS, these will be used for identification of pregnant women and neonates with positive viral RT-PCR and serology, and women with negative viral RT-PCR.

ECOSS is a database that holds surveillance data on various microorganisms (e.g. influenza virus, coronavirus) and infections reported from NHS diagnostic and reference laboratories.[30] Data on laboratory results for all SARS-CoV-2 RT-PCR tests carried out in Scotland are being collated by ECOSS and can be linked to other data sources.[30]

In sub-studies, residual sera from routine antenatal booking blood tests and 28 week gestation blood group and red cell antibody screen samples will be tested for SARS-CoV-2 antibodies. Residual sera from other blood tests conducted as part of routine (not COVID-19 related)

For peer review only

primary and secondary care, and blood donation, are also being tested for SARS-CoV-2 antibodies as part of the surveillance of the pandemic in Scotland, and any results relating to pregnant women will also be incorporated.[31] Results from these sub-studies will be linked to pregnancy records and used to determine exposure to SARS-CoV-2 by the presence of antibodies.[18]

ii) GP consultations, GP out of hours attendances, NHS24 calls, COVID-19 phone assessment hub calls, and COVID-19 clinical assessment centre attendances are linked on the EAVE II platform.[18] We will extract data on pregnant women with possible COVID-19 from GP records and a network of COVID-19 Community Hubs and Assessment Centres established by NHS Health Boards across Scotland. These provide a direct and rapid route for people with COVID-19 symptoms that have worsened or not improved after a week to seek advice and primary care. The pathway for management of patients in the community with symptoms suggestive of COVID-19 has evolved as the pandemic has progressed. In early March 2020, patients with symptoms were advised to phone their GP in hours or NHS 24 out of hours for advice. Patients requiring face to face assessment were then seen in GP surgeries or GP out of hours centres. On 17 March 2020, the Scottish Government published details of a new national patient pathway, whereby all patients with symptoms (in or out of hours) were encouraged call NHS 24 as the initial point of contact.[32] Patients thought likely to have COVID-19 were then passed to a new, dedicated NHS24 COVID-19 phone assessment hub. Those requiring face-to-face assessment in general were then seen in (or visited at home by staff from) an NHS24 COVID-19 clinical assessment centre, although pregnant women could alternatively be directed to their maternity service triage base.[32]

iii) Unscheduled Care Datamart (UCD): This links data from NHS 24, Scottish Ambulance Service, Out of Hours Primary Care, Emergency Department, Acute and Mental Health

For peer review only

admissions and Deaths to show a Continuous Unscheduled Care Pathway. Data is included in the EAVE II platform.[18] In COPS, we will use Scottish Ambulance Service incident and A&E attendance records for identification of women with possible COVID-19.[33]

iv) NRS statutory death registrations will be used for identification of any women with COVID-19 recorded as cause of death.[34]

v) SMR01, SMR02, and NRS stillbirths will be used to identify women with COVID-19 recorded as cause of admission/stillbirth.[25 27]

Datasets recording treatments, vaccination, shielding status and inclusion in other studies

i) Prescribing Information System (PIS). This includes information on all prescribed medications that are dispensed in the community in Scotland.[35] Lookback records are included in the EAVE II platform[18] and used to provide information on the presence of co-morbidities. In COPS records will also be used provide information on COVID-19 treatments given.

ii) Scottish Hospital Electronic Prescribing and Medicines Administration (HEPMA) systems are currently available within four Scottish hospitals, and data from these are linked in the EAVE II platform[18] to provide data on medications for COVID-19 administered in hospitals.[36]

iii) GP consultation records included on the EAVE II platform[18] will be used to extract information on vaccinations for SARS-CoV-2 and other viruses.

iv) We will identify pregnant women and neonates with COVID-19 in existing enhanced surveillance studies using minimal 'flag' variables from the BPSU and CO-CIN studies,[15-17] as well as other trials who can provide this data.

For peer review only - http://bmjopen.bmj.com/site/about/guidelines.xhtml

Exposure and Outcome definitions

Pregnancy, start and end dates and pregnancy outcomes

A pregnancy will be defined by presence of a record pertaining to a pregnancy. As well as identifying completed pregnancies, through a record of any pregnancy outcome, we will be able to identify ongoing pregnancies at population level, through inclusion of records from women booked for antenatal care.

The following definitions will be used for pregnancy outcomes. The codes and data sources used to identify relevant records, are described in detail in Supplementary material.

i) Ectopic pregnancy: This will include any early pregnancy loss where the pregnancy is implanted outwith the uterus

ii) Spontaneous pregnancy losses: These will be defined as miscarriage at less than 20 weeks gestation, late fetal losses at 20 - 23 weeks if there are no signs of life; and stillbirth if birth occurs at 24 weeks' gestation or more and there are no signs of life. If numbers allow, the miscarriages will be further split into the more clinically meaningful outcome categories of miscarriage at less than 14 weeks gestation; and miscarriage 14 weeks gestation and beyond.

iii) Termination of pregnancy: These will be subclassified by the grounds for termination of pregnancy of the Abortion Act 1967.[37]

iv) Live birth: The birth of a baby at any gestation with signs of life. No lower gestational limit will be used although in practice around 22 weeks gestation would be considered the lower limit at which live born babies may survive

For peer review only - http://bmjopen.bmj.com/site/about/guidelines.xhtml

Pregnancy start date will be taken as the date of conception. In pregnancies that have ended date of conception will be imputed from

"Date of conception = pregnancy end date - (the number of weeks of gestation at pregnancy end + 2 weeks)"

In ongoing pregnancies that have booked with maternity services and have a documented estimated date of delivery (EDD), date of conception will be imputed from

"Date of conception = date of antenatal booking – (gestation at booking + 2 week)"

In ongoing pregnancies without a documented EDD, date of conception will be imputed from

"Date of conception = date of last menstrual period + 2 weeks".

It is standard care for women who book with maternity services in Scotland have a first trimester ultrasound scan (usually 11-13+6 weeks gestation) to determine EDD, from which gestation is calculated. Booking takes place around 10 weeks gestation.

SARS-CoV-2 infection and COVID-19

We will use the following definitions for COVID-19.

- Confirmed COVID-19 in pregnancy will be defined as positive viral PCR for SARS-CoV-2 on a test.
- Probable COVID-19 will be defined as COVID-19 recorded on a hospital admission, stillbirth, or a maternal death record (using ICD10 codes U07.1, U07.2, B34.2, B97.2)
- Possible COVID-19 will be defined as meeting one or more of the following criteria:
    - GP consultation or GP out of hours attendance coded as possible COVID-19
    - NHS24 call coded as possible COVID-19

For peer review only

- Patient triaged to NHS24 COVID-19 phone assessment hub or clinical assessment centre
- Scottish Ambulance Service call for possible COVID-19
- A&E attendance coded as possible COVID-19
- Negative SARS-CoV-2 viral PCR test when the test was taken for clinical indications (i.e. excluding tests taken for routine testing of asymptomatic individuals)

The above definitions are hierarchical, e.g. a positive SARS-CoV-2 nucleic acid test assigns a woman to the confirmed COVID-19 group, regardless of the presence of other records.

We will use the indication for SARS-CoV-2 nucleic acid test, which is recorded in ECOSS records, to distinguish between tests taken for clinical indications and routine testing of asymptomatic individuals. If we can't distinguish between these groups, we will exclude women testing negative from both the 'case' and 'control' groups – and base our definition of possible case on presentation to various healthcare settings with relevant symptoms as described above.

SARS-CoV-2 infections during pregnancy will be identified if the event of interest (e.g. SARS-CoV-2 nucleic acid test taken, admission, unscheduled care attendance) occurs between 14 days prior to the estimated date of conception (to include women who could be viraemic periconceptually) and the end of pregnancy.

We will seek to access serological data as these become available. We will report the proportion of women with circulating IgG and/or IgM for SARS-CoV-2 and may incorporate serology results in case definitions and/or use in additional analyses as data mature. The

For peer review only

timing of exposure to/infection with SARS-CoV-2 is more difficult to ascertain from serology results than from the other indicators of (possible) infection listed above. Dates of serological testing; start and end of pregnancy; and plausible start (and, if applicable, end) of transmission of SARS-CoV-2 in Scotland will be taken into account when identifying women with exposure before, during, and after pregnancy. Seroconversion windows will also be considered for women with sequential serology results.

We have included hierarchical definitions of COVID-19 to mitigate against potential biases that may results from i) limitations of diagnostic strategy performance and ii) variation in availability of diagnostic tests. The definition of confirmed COVID-19 (women who test positive on PCR) may potentially bias results away from the null hypothesis (due to false negative PCR results). The definition of possible COVID-19 may potentially bias results towards the null hypothesis (due to false positive 'diagnoses'). We will test our hypotheses in this observational cohort across the range of assumptions allowed by hierarchical definitions. Other diagnostic and exposure categories may be added as the pandemic develops and diagnostic criteria change.

Fetal and neonatal outcomes

The following fetal and neonatal outcomes will be included.

i) Congenital anomaly (major structural anomaly as defined by EUROCAT [38] diagnosed in any pregnancy terminated at any gestation due to anomaly; miscarriage or stillbirth at ≥20 weeks; or live born baby diagnosed at <28 days of age)

For peer review only - http://bmjopen.bmj.com/site/about/guidelines.xhtml

ii) Preterm birth (<37 weeks) categorised as spontaneous or medically indicated (i.e. following induction of labour or elective Caesarean section undertaken to mitigate clinical risk)

iii) Small for gestational age (birthweight <10th centile by WHO-UK90 growth reference [39])

iv) Admission to neonatal care

v) Neonatal SARS-CoV-2 infection (currently defined as positive viral RT-PCR test on sample taken from baby aged 0-27 days, definition may be expanded to include results of serology tests as evidence and testing options accumulate)

vi) Neonatal mortality (death of a live born baby at <28 days of age)

vii) Extended perinatal mortality (stillbirth or neonatal mortality)

Maternal outcomes

We will collect data on the following maternal outcomes:

- COVID-19 disease requiring any hospital admission (defined as a patient admitted within 14 days of confirmed or probable COVID-19, or with confirmed or probable COVID-19 during admission)
- Severe COVID-19 disease requiring critical care admission or resulting in death (defined as patient admitted to critical care or dying within 28 days of confirmed or probable COVID-19, or with confirmed or probable COVID-19 during hospital admission, regardless of recorded cause of death)
- Any maternal death (defined as the death of a woman while pregnant or within 42 days of the termination of pregnancy, irrespective of the duration and site of the

For peer review only - http://bmjopen.bmj.com/site/about/guidelines.xhtml

pregnancy, from any cause related to or aggravated by the pregnancy or its management but not from accidental or incidental causes)

Population characteristics and confounding factors

A number of maternal and pregnancy characteristics will be collected that could be potential confounders or effect modifiers.

i) Demographics including age band and socioeconomic status determined by the Scottish Index of Multiple Deprivation (SIMD) classification of material deprivation.[40] SIMD Quintiles 1 through 5 refer to the small geographical areas (data zones) each containing 20% of the Scottish population, with quintile 1 indicating the most deprived areas. The SIMD is a combination of 38 indicators of the following seven domains: income, employment, health, education, housing, geographical access to services, and crime. We will also include urban/rural status of maternal residence based on the urban/rural 8 fold classification (UR8)[41] where 1 is assigned to large urban areas and 8 is assigned to remote rural areas. We recognise ethnicity to be a complex indicator variable related to sociodemographic factors, health systems use, pregnancy and health outcomes and genetics. We will explore the possibility of including self-reported maternal ethnicity although missing data may preclude this.

ii) Clinical at-risk groups of individuals with certain underlying medical conditions thought to increase risk of COVID-related complications. The following clinical at-risk conditions are identified in the EAVE II platform[18]: a) chronic respiratory disease; b) chronic heart disease; c) chronic liver disease; d) chronic kidney disease; e) chronic liver disease; f) chronic neurological disease; g) Diabetes; h) Conditions or medications causing impaired immune function; i) Asplenia or dysfunction of spleen and j) body mass index (BMI). In addition, we will link pregnancy records with the shielded patient list included in the EAVE II platform

For peer review only

(those with extremely high risk of severe manifestation of SARS-CoV-2 infection, and hence advised by Scottish Government to 'shield' during the pandemic). We will re-categorise clinical risks for the pregnant population as i) Diabetes (Type I; Type II; Other pre-pregnancy; Gestational Diabetes); ii) clinically vulnerable risk group (for whom seasonal influenza vaccination is recommended outwith pregnancy); iii) clinically extremely vulnerable risk group (those advised to 'shield' during the pandemic);[42] and iv) no clinical at-risk condition. BMI, which can be associated with adverse pregnancy outcomes as well as COVID-disease will be included separately, with pre-pregnancy BMI or BMI at antenatal booking categorised as Underweight, Normal, Overweight, Obese and Severely Obese according to WHO definitions.[43] Other categorisation will be considered depending on numbers of pregnant women with these conditions, and emergence of patterns of risk for COVID-19 disease.

iii) Smoking status in pregnancy will be presented into the following four categories: Current smoker, non-smoker, ex-smoker and not recorded. Smoking status will be taken from booking record and/or GP records.

iv) Obstetric characteristics (SMR02) will include previous pregnancies, plurality (number of babies), drug and alcohol use, antenatal steroid administration, mode of birth or management of pregnancy loss.

v) Clustering of outcomes by the 14 different Maternal NHS Board areas of residence.

Statistical analysis

General Approach

Baseline characteristics of all study participants will be described in relation to presence and absence of confirmed, probable or possible COVID-19 and outcomes of interest. Mean, median

For peer review only

and proportions, together with a measure of dispersion will be provided where appropriate to describe differences between the various groups of interest based on the nature of each variable. Missing data will be provided for each variable. Two-tailed hypotheses tests will be used for all study's outcomes, with 95% confidence intervals presented to show precision of estimates, and p values reported. All analyses will be carried out using the R statistical programming language. We do not propose to make any formal statistical adjustment for the multiple comparisons as the principal aim of the study is to estimate the effect of COVID-19 infection on pregnancy outcomes. The estimated effects and 95% confidence intervals will be reported for the range of outcomes. However, a caveat will be clearly expressed regarding the dangers of over interpreting these data, given the multiple outcomes used, particularly if it transpires that conflicting results are obtained from the differing outcome measures. The approach to imputing estimated date of conception when gestation is missing on records indicating pregnancy status is detailed in Supplementary material 1. Missing data are otherwise not anticipated to be a substantial problem (and hence imputation techniques are not anticipated) but this will be confirmed once initial data extracts are available, and our approach to handling missing data will be confirmed prior to analysis.

Analyses will be updated monthly, providing results for sequential months, and also information on the cumulative risk of COVID-19 as women progress through their pregnancies. Simple smoothing techniques such as rolling averages will be used to facilitate presentation and interpretation of findings. We will also present our results as proportions of COVID-19 infection, together with confidence intervals based upon the Wilson method. We will describe the temporal changes in the proportion using cumulative risk models. Covariates such as the trimester of pregnancy, age of the mother and deprivation will also be

For peer review only

included with a view to estimating the potential effects of these variables on the risk of COVID-19 infection

If/when numbers of cases allow, we will examine incidence of COVID-19, and report outcomes, in subgroups including by maternal age band, SIMD deprivation quintile, maternal NHS Board area of residence and maternal comorbidity status. We may assess whether findings are robust to more stringent or emerging definitions of confirmed and suspected infection, in sensitivity analyses. Other sensitivity and subgroup analyses may be indicated by initial findings. We will clearly state which analyses were pre-specified and which were post-hoc.

Incidence of SARS-CoV-2 and COVID-19 in the pregnant population

We will perform descriptive analysis of the number of cases over the total number of pregnancies i.e. how many pregnant women have had confirmed, probable or possible COVID-19/ total number of pregnant women. Where timing of infection is known, we will describe incidence of SARS-CoV-2 infection by trimester of exposure - first trimester (0-13 weeks gestation) second trimester (14-27 weeks gestation); third trimester (≥28 weeks gestation)[44]; with denominators consisting of ongoing pregnancies in each trimester.

Associations between COVID-19 and adverse pregnancy, neonatal and maternal outcomes.

Initially we will perform a descriptive analysis comparing pregnancy outcomes in women with and without i) confirmed; ii) probable and iii) possible COVID-19. In order to create appropriate comparison groups, for each woman with COVID-19, we will identify ten women without COVID-19, with an ongoing pregnancy matched on gestation of diagnosis,

For peer review only - http://bmjopen.bmj.com/site/about/guidelines.xhtml

and additionally matched on maternal age and maternal deprivation level. We will explore the need to match additional parameters such as NHS Board.

Occurrence of the outcomes of interest will be compared in women with and without COVID-19 using simple descriptive statistics (e.g. 95% confidence interval for the difference in proportions, generated using methods which accommodate proportions close to zero) and visualised appropriately.

If/when sufficient cases of COVID-19 among pregnant women accrue, and the univariable comparisons described above suggest that outcomes differ between women with and without SARS-2-CoV or COVID-19, formal modelling will be undertaken to quantify the impact of infection on outcomes, adjusting appropriately for confounding. We will use direct acyclical graphs (DAGs) to identify which factors to adjust for to mitigate for confounding. Appropriate methods that accommodate the competing risk and time to event nature of pregnancy outcomes (example event history analysis and/or multistate modelling) will be used.

Association of SARS-CoV-2 in neonates with maternal COVID-19

We will use summary statistics to describe neonatal SARS-CoV-2 (currently defined as positive viral PCR for SARS-CoV-2 on sample taken from a baby aged 0-27 days old) by presentation of COVID-19 in the mother in different time periods (apparent onset of maternal illness >14 days prior to birth; 14 days prior to birth - date of birth; day 1 - 13 following birth; day 14 - 27 following birth).

Proportion of pregnant women and neonates with COVID-19 or SARS-CoV-2 that are included in relevant other enhanced surveillance studies (BPSU, CO-CIN)

For peer review only - http://bmjopen.bmj.com/site/about/guidelines.xhtml

We will use summary statistics to describe the number and proportion of cases included in the external surveillance studies, and any factors associated with inclusion e.g. hospital admission status and NHS Board area of residence.

Creation of a platform to assess the safety and effectiveness of any new or existing prophylactic or therapeutic interventions and assessment of childhood outcomes after pregnancy exposure to COVID-19

We will use summary statistics to describe the treatments and prophylactic interventions used in pregnancy. We plan future linkage of data within COPS with child health and education data to allow assessment of long-term outcomes.

Sample size

There are approximately 50,000 live births in Scotland per year, 13,000 terminations of pregnancy, 5,000 miscarriages managed in hospital and 200 stillbirths. The estimated number of women in the population who are pregnant at any one time is approximately 42,000.

We cannot influence the number of women with confirmed, probable or possible COVID-19 available for analysis hence sample size calculations will not be performed. We will report the precision with which we are able to estimate any association between COVID-19 and the outcomes of interest using confidence intervals as appropriate. An approximate estimate of the expected number of confirmed COVID-19 cases in pregnant women from March to May 2020 is presented in Table 2. It is likely that there will be further confirmed or probable cases in pregnant women identified through PCR testing processed through UK Government laboratories and clinical diagnoses on discharge (or possibly stillbirth or maternal death)

For peer review only - http://bmjopen.bmj.com/site/about/guidelines.xhtml

records. In addition, it is likely that there will be considerably more possible cases among pregnant women based on the range of data sources listed above.

ETHICS AND DISSEMINATION

COPS is a sub-study of EAVE II, using unconsented data, which is covered by National Research Ethics Service Committee, South East Scotland 02 approval reference REC 12/SS/0201: SA 2 and Public Benefit and Privacy Panel approval reference 2021-0116. Public Health Scotland and the Chief Medical Officer for Scotland are both (independent) data controllers for the national AAS database of termination of pregnancy notifications, thus the Chief Medical Officer has been informed of the intended use of AAS records for this study.

The results of monthly analyses summarising the incidence of COVID-19 in pregnant women, and outcomes seen in women with COVID-19 and pregnant controls, will be reported through the Public Health Scotland COVID-19 enhanced surveillance cell to the Scottish Government's Chief Medical Officer's COVID-19 Advisory Group. Any results of formal modelling of outcomes that is undertaken will be reported through the same route. Results reported through this route may be provided as management information (i.e. without application of statistical disclosure control restrictions) as appropriate.

Results will also be submitted for peer reviewed academic publication and presented at international conferences. All results put into the public domain will be subject to statistical disclosure control according to usual Public Health Scotland processes. Meta-data produced in this study will also become available to Health Data Research UK (HDRUK) Gateway. Strengthening the Reporting of Observational Studies in Epidemiology (STROBE)

For peer review only - http://bmjopen.bmj.com/site/about/guidelines.xhtml

guidance[45] and Reporting of studies Conducted using Observational Routinely-collected Data (RECORD) guidance[46] will be used to guide transparent reporting**.**

AUTHOR CONTRIBUTIONS

SJS, RW, CR and AS contributed to the conception of the study. SJS, DM, EV, CRS, HRS, UA, CM, LH, JD, LR, CR, AS, and RW contributed to the study design. SJS, DM, EV, CRS, HRS, UA, CM, LH, JD, LR, CR, AS, and RW authors contributed to drafting the protocol. SJS, DM, EV, CRS, HRS, UA, CM, LH, JD, LR, CR, AS, and RW revised the manuscript for important intellectual content. SJS, DM, EV, CRS, HRS, UA, CM, LH, JD, LR, CR, AS, and RW gave final approval of the version to be published.

ACKNOWLEDGMENTS

The authors thank and acknowledge the wider EAVE II team for their support for this protocol.

FUNDING

EAVE II funded by the Medical Research Council (MR/R008345/1) with the support of BREATHE - The Health Data Research Hub for Respiratory Health [MC_PC_19004], which is funded through the UK Research and Innovation Industrial Strategy Challenge Fund and delivered through Health Data Research UK. Additional support has been provided through the Scottish Government DG Health and Social Care. COPS receive additional funding from Tommy's charity (1060508; SC039280). SJS is supported by Wellcome Trust (209560/Z/17/Z)

COMPETING INTERESTS

For peer review only - http://bmjopen.bmj.com/site/about/guidelines.xhtml

None declared.

DATA STATEMENT

Public Health Scotland and the Chief Medical Officer of Scotland are data controllers of the data used within the study. We will not be able to share data for the study as we are not the data controllers. All results put into the public domain will be subject to statistical disclosure control according to usual processes. Meta-data produced in this study will be made available to Health Data Research UK (HDRUK) Gateway. Applications to use the datasets included in the study can be made via https://www.informationgovernance.scot.nhs.uk/pbpphsc/

REFERENCES

1. Coronavirus (COVID-19) Infection in Pregnancy. 2020. Royal College of Obstetricians and Gynaecologists. Available from: https://www.rcog.org.uk/globalassets/documents/guidelines/2020-06-18-coronavirus-covid-19-infection-in-pregnancy.pdf. Accessed 18th July 2020.

2. Guidance on shielding and protecting people who are clinically extremely vulnerable from COVID-19. Public Health England. 2020. Available from: https://www.gov.uk/government/publications/guidance-on-shielding-and-protecting-extremely-vulnerable-persons-from-covid-19/guidance-on-shielding-and-protecting-extremely-vulnerable-persons-from-covid-19#who-is-clinically-extremely-vulnerable Accessed 14th July 2020

3. Di Mascio D, Khalil A, Saccone G, et al. Outcome of Coronavirus spectrum infections (SARS, MERS, COVID 1 -19) during pregnancy: a systematic review and meta-analysis. Am J Obstet Gynecol MFM 2020:100107.

For peer review only - http://bmjopen.bmj.com/site/about/guidelines.xhtml

4. If You Are Pregnant, Breastfeeding, or Caring for Young Children. Centers for Disease Control and Prevention. 2020. Available from: https://www.cdc.gov/coronavirus/2019-ncov/need-extra-precautions/pregnancy-breastfeeding.html?CDC_AA_refVal=https%3A%2F%2Fwww.cdc.gov%2Fcoronavirus%2F2019-ncov%2Fhcp%2Fpregnant-women-faq.html Accessed 14th July 2020

5. Huntley BJF, Huntley ES, Di Mascio D, et al. Rates of Maternal and Perinatal Mortality and Vertical Transmission in Pregnancies Complicated by Severe Acute Respiratory Syndrome Coronavirus 2 (SARS-Co-V-2) Infection: A Systematic Review. Obstet Gynecol 2020

6. Racicot K, Mor G. Risks associated with viral infections during pregnancy. J Clin Invest 2017;127(5):1591-99.

7. Coyne CB, Lazear HM. Zika virus - reigniting the TORCH. Nat Rev Microbiol 2016;14(11):707-15.

8. Zheng Q-L, Duan T, L-P. J. Single-cell RNA expression profiling of ACE2 and AXL in the human maternal–Fetal interface. Reprod Dev Med [Internet] 2020;2020 Mar 25 4(1):7.

9. Pique-Regi R, Romero R, Tarca AL, et al. Does the human placenta express the canonical cell entry mediators for SARS-CoV-2? bioRxiv 2020 May 18;2020.05.18.101485.

10. Hosier H, Farhadian S, Morotti R, et al. SARS-CoV-2 Infection of the Placenta. medRxiv 2020 May 12;2020.04.30.20083907.

11. Baud D, Greub G, Favre G, et al. Second-Trimester Miscarriage in a Pregnant Woman With SARS-CoV-2 Infection. JAMA 2020 doi: 10.1001/jama.2020.7233 [published Online First: 2020/05/01]

12. Mother-to-child transmission (MTCT). University of Birmingham COVID-19 in Pregnancy (PregCOV-19LSR). 2020. Available from:

For peer review only

https://www.birmingham.ac.uk/research/who-collaborating-centre/pregcov/about/mother-to-child-transmission.aspx Accessed 14th July 2020

13. Dong L, Tian J, He S, et al. Possible Vertical Transmission of SARS-CoV-2 From an Infected Mother to Her Newborn. JAMA 2020 doi: 10.1001/jama.2020.4621 [published Online First: 2020/03/28]

14. Zeng H, Xu C, Fan J, et al. Antibodies in Infants Born to Mothers With COVID-19 Pneumonia. JAMA 2020 doi: 10.1001/jama.2020.4861 [published Online First: 2020/03/28]

15. Multisystem inflammatory syndrome, Kawasaki disease and toxic shock syndrome. British Paediatric Surveillance Unit. 2020. Available from: https://www.rcpch.ac.uk/work-we-do/bpsu/study-multisystem-inflammatory-syndrome-kawasaki-disease-toxic-shock-syndrome Accessed 14th July 2020

16. Neonatal complications of coronavirus disease (COVID-19). British Paediatric Surveillance Unit. 2020. Available from: https://www.rcpch.ac.uk/work-we-do/bpsu/study-neonatal-complications-coronavirus-disease-covid-19 Accessed 14th July 2020

17. COVID-19 database (CO-CIN). International Severe Acute Respiratory and Emerging Infection Consortium (ISARIC). 2020. Available from: https://isaric.tghn.org/covid-19-clinical-research-resources Accessed 14th July 2020

18. Simpson CR, Robertson C, Vasileiou E, et al. Early Pandemic Evaluation and Enhanced Surveillance of COVID-19 (EAVE II): protocol for an observational study using linked Scottish national data. BMJ Open 2020;10(6):e039097.

19. Simpson CR, Beever D, Challen K, et al. The UK's pandemic influenza research portfolio: a model for future research on emerging infections. Lancet Infect Dis 2019;19(8):e295-e300.

20. Simpson CR, Thomas BD, Challen K, et al. The UK hibernated pandemic influenza research portfolio: triggered for COVID-19. Lancet Infect Dis 2020;20(7):767-69.

For peer review only - http://bmjopen.bmj.com/site/about/guidelines.xhtml

For peer review only

21. Da Silva Filipe A, Shepherd J, Williams T, et al. Genomic epidemiology of SARS-CoV-2 spread in Scotland highlights the role of European travel in COVID-19 emergence. medRxiv 2020;20124834.

22. Simpson CR, Ritchie LD, Robertson C, et al. Vaccine effectiveness in pandemic influenza - primary care reporting (VIPER): an observational study to assess the effectiveness of the pandemic influenza A (H1N1)v vaccine. Health Technol Assess 2010;14(34):313-46.

23. Public Health Scotland. Births in Scotland 2018-2019 2019. Available from: https://www.isdscotland.org/Health-Topics/Maternity-and-Births/Births/ Accessed 14th July 2020

24. Notification of Abortion Statistics (AAS). Public Health Scotland. 2020. Available from: https://www.ndc.scot.nhs.uk/National-Datasets/data.asp?SubID=64. Accessed 14th July 2020

25. Stillbirths and Infant Deaths. National Records of Scotland. 2020. Available from: https://www.nrscotland.gov.uk/statistics-and-data/statistics/statistics-by-theme/vital-events/deaths/deaths-background-information/stillbirths-and-infant-deaths Accessed 14th July 2020

26. Vital Events – Births. National Records of Scotland. 2020. Available from: https://www.nrscotland.gov.uk/statistics-and-data/statistics/statistics-by-theme/vital-events/births

27. Scottish Morbidity Record Type. Public Health Scotland. 2020. Available from: https://www.ndc.scot.nhs.uk/Data-Dictionary/SMR-Datasets/Episode-Management/SMR-Record-Type/ Accessed 14th July 2020

28. Scottish Birth Record. Public Health Scotland. 2020. Available from: https://www.isdscotland.org/Products-and-Services/Scottish-Birth-Record/ Accessed 14th July 2020

For peer review only

For peer review only - http://bmjopen.bmj.com/site/about/guidelines.xhtml

29. Scottish Intensive Care Society Audit Group Public Health Scotland. 2020. Available from: https://www.sicsag.scot.nhs.uk/index.html Accessed 14th July 2020

30. Communication of Surveillance in Scotland (ECOSS). Health Protection Scotland. 2020. Available from: https://www.hps.scot.nhs.uk/data/ Accessed 14th July 2020

31. Corona Virus 19 NHS Greater Glasgow and Clyde. 2020. Available from: https://www.nhsggc.org.uk/about-us/professional-support-sites/laboratory-medicine/laboratory-disciplines/microbiology-and-virology/west-of-scotland-specialist-virology-centre/# Accessed 14th July 2020

32. Community pathway for managing Covid-19 in Scotland- Delay phase. Scottish Government Heath and Social Care Directorates. 2020 Available from: https://www.sehd.scot.nhs.uk/publications/DC20200317Covid-19.pdf Accessed 14th July 2020

33. Urgent Care Data Mart (UCD) – Background Paper. Public Health Scotland. 2017. Available from: https://www.isdscotland.org/Health-Topics/Emergency-Care/Patient-Pathways/UrgentCareDataMartBackgroundPaper_20171002.pdf Accessed 14th July 2020

34. Vital Events – Deaths. National Records of Scotland. 2020. Available from: https://www.nrscotland.gov.uk/statistics-and-data/statistics/statistics-by-theme/vital-events/deaths Accessed 14th July 2020

35. Prescribing Information System (PIS). Public Health Scotland. 2020. Available from: https://www.ndc.scot.nhs.uk/National-Datasets/data.asp?SubID=9 Accessed 14th July 2020

36. ehealth. HEPMA [Available from: https://www.ehealth.scot/case-studies/hepma/.

37. Abortion Act 1967. UK Government. 1967. Available from: http://www.legislation.gov.uk/ukpga/1967/87/contents Accessed 14th July 2020

38. EUROCAT Guide 1.4: Instruction for the registration of congenital anomalies. In: EUROCAT Central Registry, ed. University of Ulster, 2013.

For peer review only

39. Wright CM, Booth IW, Buckler JM, et al. Growth reference charts for use in the United Kingdom. Arch Dis Child 2002;86(1):11-4.

40. Scottish Index of Multiple Deprivation. Scottish Government. 2020. Available from: https://www.gov.scot/collections/scottish-index-of-multiple-deprivation-2020/ Accessed 14th July 2020

41. Scottish urban/rural 8 fold classification. Scottish Government. 2020. Available from: https://www2.gov.scot/Topics/Statistics/About/Methodology/UrbanRuralClassification Accessed 14th July 2020

42. COVID-19 - search criteria for highest risk patients for shielding. Health Protection Scotland. 2020. Available from: https://www.hps.scot.nhs.uk/web-resources-container/covid-19-search-criteria-for-highest-risk-patients-for-shielding/ Accessed 14th July 2020

43. WHO. Clinical Guidelines on the Identification, Evaluation, and Treatment of Overweight and Obesity in Adults--The Evidence Report. National Institutes of Health Obes Res 6 Suppl 2:51S-209S 1998

44. How your fetus grows during pregnancy. American College of Obstetrics and Gynaecology. Available from: https://www.acog.org/patient-resources/faqs/pregnancy/how-your-fetus-grows-during-pregnancy Accessed 11th September 2020

45. von Elm E, Altman DG, Egger M, et al. Strengthening the Reporting of Observational Studies in Epidemiology (STROBE) statement: guidelines for reporting observational studies. BMJ 2007;335(7624):806-8.

46. Benchimol EI, Smeeth L, Guttmann A, et al. The REporting of studies Conducted using Observational Routinely-collected health Data (RECORD) statement. PLoS Med 2015;12(10):e1001885.

For peer review only

47. COVID-19 in Pregnancy. UKOSS University of Oxford.2020. Available from: https://www.npeu.ox.ac.uk/ukoss/current-surveillance/covid-19-in-pregnancy Accessed 14th July 2020

48. Pregnancy And Neonatal outcomes for women with COVID-19 (PAN-COVID). Imperial College London. 2020. Available from: https://pan-covid.org Accessed 14th July 2020

49. PERICOVID. Public Health England. 2020. Available from: https://www.pericovid.com Accessed 14th July 2020

50. Coronavirus (COVID-19) data. Public Health Scotland. 2020. Available from: https://publichealthscotland.scot/our-areas-of-work/sharing-our-data-and-intelligence/coronavirus-covid-19-data/ Accessed 14th July 2020

For peer review only

TABLES

Table 1: UK surveillance studies on COVID-19 in pregnant women and their babies

Name of study	Institution	Inclusion	Reporting by	Consent required	Likely coverage in Scotland
COVID-19 in Pregnancy [47]	UK Obstetric Surveillance System study (UKOSS)	Any women admitted to hospital in the UK with confirmed COVID-19 at any stage of pregnancy	Front-line clinicians	No	High
Pregnancy And Neonatal outcomes for women with COVID-19 (PAN-COVID) [48]	National Institute of Healthcare Research (NIHR) Imperial Biomedical Research Centre	Women who have suspected or confirmed COVID-19 at any stage during pregnancy and their babies	Front-line clinicians	Yes	Unknown as yet
Clinical Characterisation Protocol Tier 0	The International Severe Acute	Any patient admitted participating	Reporting is by research nurses	No	Low but may increase

For peer review only - http://bmjopen.bmj.com/site/about/guidelines.xhtml

study (CO-CIN) [17]	Respiratory and emerging Infection Consortium (IASRIC)	hospitals in the UK with confirmed COVID-19			
Neonatal complications of coronavirus disease (COVID-19) [17]	British Paediatric Surveillance Unit (BPSU)	All babies born to mothers with COVID-19 who are admitted to neonatal care (whether the baby has COVID-19 or not) and all babies with confirmed COVID-19 in the neonatal period.	Front-line clinicians.	No	High
Multisystem inflammatory syndrome, Kawasaki disease and toxic	British Paediatric Surveillance Unit (BPSU)	All children less than 16 years old (including neonates) with	Front-line clinicians	No	High

For peer review only - http://bmjopen.bmj.com/site/about/guidelines.xhtml

shock syndrome[15]		multisystem inflammatory syndrome due to SARS-CoV-2 infection or otherwise unexplained.			
Understanding COVID-19 infection in women and their babies (periCOVID)[49]	Public Health England and St George's University London	Any pregnant woman with confirmed COVID-19 infection from 24 weeks gestation in England	Clinicians/research midwives and nurses	Yes	None

For peer review only

Table 2: Estimated number of confirmed COVID-19 cases March to May 2020 in pregnant women in Scotland

	Total number of individuals testing positive (PCR) for SARS-CoV-2 (NHS labs only)	Women aged 15-44 years testing positive (PCR) for SARS-CoV-2 (NHS labs only)	Estimated number of pregnant women testing positive (PCR) for SARS-CoV-2 (NHS labs only)**
March 2020	≈2000	≈333*	≈17
April 2020	≈9000	≈1500*	≈75
May 2020	≈4000	≈667*	≈33
Total	≈15000	≈2500	≈125

* Assuming the distribution over time for this age/sex group is the same as for all tests, as age/sex breakdown only available from published information[50] for the total

** Assuming that around 5% of the female population aged 15-44 is pregnant at any one time, and that incidence of COVID-19 is the same in pregnant and non-pregnant women

For peer review only

FIGURE LEGENDS

Figure 1: Overview of data linkage for the Covid-19 in Pregnancy in Scotland (COPS) study

For peer review only

For peer review only - http://bmjopen.bmj.com/site/about/guidelines.xhtml

Figure 1: Overview of data linkage for the Covid-19 in Pregnancy in Scotland (COPS) study

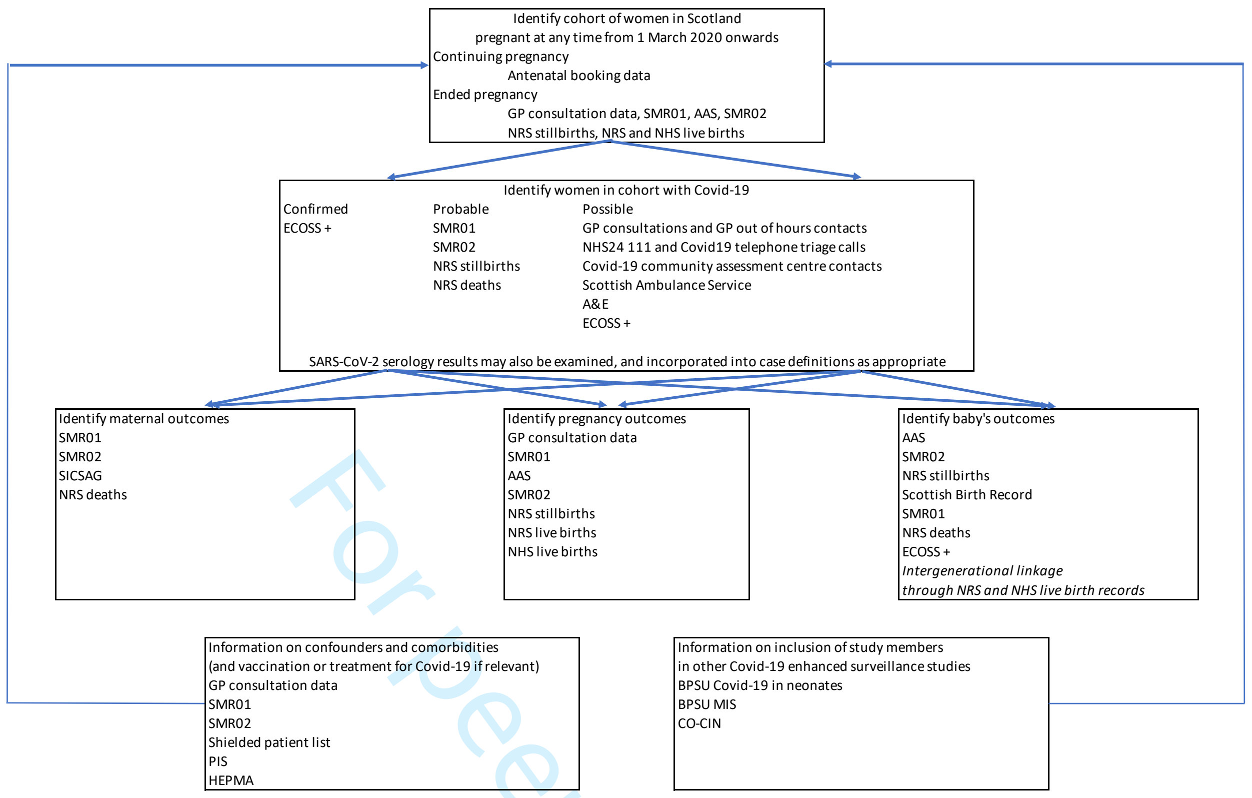

Supplementary Material: Additional information on identifying pregnant women, and associated start and end of pregnancy dates, from national data sources

National data sources identifying end of pregnancy events

There are three possible outcomes for any pregnancy

Pregnancy outcome	Comments
Spontaneous loss	'Miscarriage' at <24w (here taken to include ectopic pregnancies, although mechanisms underlying spontaneous miscarriage and ectopic pregnancy differ)<br>(Sometime 'late fetal loss' at 20-23w as a subset of miscarriages)<br>'Stillbirth' at ≥24w
Termination of pregnancy	Legal at <24w under Grounds C and D of the Abortion Act 1967<br>Legal at any gestation under Grounds A, B, E, F, G
Live birth	No lower gestational limit although in practice around 22w would be considered the lower limit at which live born babies may survive

For peer review only

Various national records may be returned following these end of pregnancy events, as summarised below

National record	Description	Pregnancy outcomes identified	Coding to identify relevant records
Identifying spontaneous pregnancy losses			
SMR01	Record of day case or inpatient admission to any general unit (excluding neonatal, maternity, and mental health care), including admissions under gynaecology specialty	Will identify early (first trimester) spontaneous losses managed in hospital in most Board areas	ICD10:<br>O00 (ectopic pregnancy)<br>O01 (hydatidiform mole)<br>O02 (missed miscarriage)<br>O03, O05, O06 (spontaneous miscarriage), all .5-.9
OR<br>SMR02	Record of day case or inpatient admission to a maternity unit, including admissions under obstetrics or midwifery specialties	Will identify early (first trimester) spontaneous losses managed in hospital in some Board areas<br>Will identify later (second and third trimester) spontaneous losses managed in hospital in all areas	Miscarriages<br>Condition on discharge=2 (aborted)<br>Type of abortion=1, 2, 3, 6, 8, 9 (spontaneous)<br><br>Stillbirths<br>Condition on discharge=3 (delivered)<br>Outcome of pregnancy=2 (stillbirth)
AND/OR<br>NRS stillbirths	Record of statutory registration of a stillbirth (baby born at ≥24w showing no signs of life)	Will identify spontaneous stillbirths	ICD10:<br>P96.4 not recorded
Identifying terminations of pregnancy			
AAS	Record of statutory notification of a termination of pregnancy	Should identify all terminations of pregnancy but known under-notification of later ToPs done for fetal anomaly from some maternity units	

For peer review only

AND/OR<br>SMR02	As above	Will identify later ToPs done for fetal anomaly in maternity units	Condition on discharge=2 (aborted)<br>Type of abortion=4 (ToP)
AND/OR<br>NRS stillbirths	As above	Will identify the small number of stillbirths following a termination of pregnancy	ICD10:<br>P96.4 recorded in any position
Identifying live births			
SMR02	As above	Will identify live births occurring in hospital<br>SMR02 returns were enabled to cover home (as well as in hospital) births from Apr 2019, and coverage of home births should have been mandatory from Oct 2019, however technical difficulties mean that home births are still (as at July 2020) not recorded on SMR02 in most Boards	Condition on discharge=3 (delivered)<br>Outcome of pregnancy=1, 3, 4, 5 (live birth)
AND/OR<br>NRS live births	Record of statutory registration of a live birth (live born baby at any gestation)	Usually identifies all live births however statutory registration of live births was suspended from 23 March to 28 June 2020 inclusive when registrar offices closed<br>The only babies being registered during that period were those that subsequently die: this was done remotely along with the death registration to avoid parents having to register the birth in person later<br>A catch up programme of live birth registrations started on 29 June 2020	

For peer review only

AND/OR<br><br>NHS live birth notifications	Notification of live births from NHS Board maternity units to child health administration departments<br><br>This notification allows a record to be created for the child on the national child health information system: this in turn ensures the child is called for immunisations and child health reviews	As NRS live birth registration was suspended in March – June 2020 due to COVID-19 (see above), PHS has recently developed a new data extraction from the national child health information system of birth notification data<br><br>This will identify all live births known to NHS maternity services from Aug 2019 onwards<br><br>A small number of babies who die very soon after birth (before that day's notification data has been sent) will not be included as these babies do not need to be notified for ongoing care, however they will be covered by NRS registration as noted above	

It is possible that the same woman/pregnancy may have multiple records giving conflicting information on the outcome of the pregnancy.

In general, if any record indicates a termination of pregnancy, this should be taken as the outcome.

If an NRS stillbirth record is available for a baby but the corresponding SMR02 record indicates the baby was live born, this should be taken as a stillbirth.

For peer review only

The relevant gestation and date of event information in the various records, and how to deal with missing gestation information, is summarised below

National record	Gestation information available	Date of event information available	Dealing with missing gestation information (due to not recorded on that record, missing, or recorded but unfeasible)
SMR01	None	Date of admission<br>Date of discharge	Assume 12 weeks gestation at date of admission
SMR02	Gestation in completed weeks at end of pregnancy available on records where Condition on discharge=2 or 3 (aborted or delivered)	Date of admission<br>Date of discharge<br>Date of delivery on records where Condition on discharge=3 (delivered)	Miscarriage records with missing gestation, assume 12 weeks gestation at date of admission<br>ToP records with missing gestation (and not available from AAS), assume 16 weeks gestation at date of admission<br>Stillbirth delivery records with missing gestation (and not available from NRS), assume 32 weeks gestation at date of delivery<br>Live birth delivery records with missing gestation, assume 40 weeks gestation at date of delivery
NRS stillbirths	Gestation in completed weeks at date of stillbirth available	Date of stillbirth	Assume 32 weeks gestation at date of delivery (if not available from SMR02)
AAS	Gestation in completed weeks at date of termination available	Date of termination (date of administration of antiprogesterone for medical ToPs)	Assume 10 weeks gestation at date of termination (if not available from SMR02)
NRS live births	None	Date of birth	Assume 40 weeks gestation at date of birth (if not available from SMR02)

For peer review only

NHS live birth notifications	Gestation in completed weeks at date of birth available<br>(Although note this data has not been used before by PHS so will require checking before use)	Date of birth	Assume 40 weeks gestation at date of birth (if not available from SMR02)

For peer review only

The time lag inherent in the different data returns is summarised below

National record	Time lag inherent in data source
SMR01	Records should be returned to PHS within 6 weeks of patient's discharge (in practice sometimes longer)<br>Monthly batches (all records received to that point) are then uploaded to the analysis platform (SMRA) around the middle of each month<br>Records are CHI seeded as they are uploaded to the analysis platform (and CHI seeding is refreshed monthly thereafter to pick up any previously unseeded records)<br>CHI seeding usually complete on first attempt<br>So: records relating to discharges in Jan XX should be available for linkage and analysis within PHS in mid Apr XX (2.5 month lag)
SMR02	Records should be returned to PHS within 6 weeks of patient's discharge (in practice sometimes longer)<br>Monthly batches (all records received to that point) are then uploaded to the analysis platform around the middle of each month<br>Records are CHI seeded as they are uploaded to the analysis platform (and CHI seeding is refreshed monthly thereafter to pick up any previously unseeded records)<br>Maternal CHI seeding usually complete on first attempt<br>Baby CHI seeding usually complete on second attempt<br>So: as linkage of SMR02 records is generally through maternal CHI, records relating to discharges in Jan XX should be available for linkage and analysis within PHS in mid Apr XX (2.5 month lag)

For peer review only

NRS stillbirths	Registration required within 21 days of birth<br>Data transferred by NRS to PHS weekly<br>Monthly batches (stillbirths registered up to the end of the previous month) are then uploaded to the analysis platform around the middle of each month<br>In parallel, records are sent to NHSCR monthly for seeding of maternal CHI<br>As seeded records are returned from NHSCR, the CHIs are added to the records on the analysis platform<br>So: records relating to stillbirths occurring in Jan XX should be available for linkage and analysis within PHS in mid May XX (3.5 month lag)<br>(Note: almost all stillbirths will have an SMR02 record so can be identified and linked with 2.5 month lag)
AAS	Notification to CMO required within 7 days of termination<br>Records forwarded to PHS and entered into AAS system (includes automated CHI seeding) within 6 weeks of date of termination<br>So: records relating to terminations occurring in Jan XX should be available for linkage and analysis within PHS in mid Mar XX (1.5 month lag)

For peer review only

NRS live births	Registration required within 21 days of birth<br>Data transferred by NRS to PHS weekly<br>Monthly batches (live births registered up to the end of the previous month) are then uploaded to the analysis platform around the middle of each month<br>Records are seeded with baby CHI as they are uploaded to the analysis platform (and CHI seeding is refreshed monthly thereafter to pick up any previously unseeded records)<br>Baby CHI seeding usually complete on second attempt<br>In parallel, monthly batches are seeded with the mother's CHI by bespoke linkage to SMR02 after a 6 month lag (i.e. records for births in Jan XX and matched against SMR02 in Jul XX)<br>Records with no maternal CHI found are then matched against the full CHI database<br>Residual records with still no maternal CHI are then sent to NHSCR in monthly batches<br>So: as linkage of NRS live birth records generally requires both maternal and baby CHI (to allow intergenerational linkage), records relating to births in Jan XX should be available for linkage and analysis within PHS in mid Oct XX (8.5 month lag)<br>(Note: all live births from Aug 2019 onwards will have a birth notification record available so can be identified and linked with a 1 month lag)
NHS live birth notifications	Live births are notified to the NHS Board child health admin department within 1 working day of date of birth and are keyed into the national child health info system promptly (same or subsequent day)<br>PHS extracts notification data (including baby's CHI) from the national child health info system weekly<br>Maternal CHI is then seeded onto the data extracts weekly<br>So: records relating to births in Jan XX should be available for linkage and analysis within PHS in Feb XX (1 month lag)

For peer review only

National data sources identifying continuing pregnancies as early as possible

As part of the response to COVID-19, PHS has established a new national data return providing information on women booking for antenatal care. This will allow us to identify pregnant women before the end of their pregnancy, and hence monitor SARS-CoV-2 infections occurring in pregnant women in closer to real time. Further information on this data source is provided below.

Data items being requested in the new data feed include

- Maternal CHI
- Mother's Forename, Surname, Date of Birth, and Postcode in case CHI is missing and needs to be appended
- Date of Booking
- Gestation at booking
- Date of Last Menstrual Period (in case gestation is missing)

PHS has asked NHS Boards to provide an initial submission of historic data on all women booking from 1 April 2019, then subsequent weekly updates. The weekly updates will give information on women who have booked in the most recent week, and also update any records relating to the previous 2 weeks if those have changed since the previous submission. The current assumption is that this data will be submitted with maternal CHI complete, hence additional lag for CHI seeding will not be required but this is being kept under review.

This dataset will identify all women booking for NHS antenatal care. The method of providing booking services has changed in many areas due to COVID-19, with many Boards now providing the initial booking appointment remotely, with the woman subsequently attending in person for her initial ultrasound scan and blood tests[1]. To ensure that the dataset allows us to identify pregnant women as early as possible in their maternity care journey, the 'booking' event that is captured in the above dataset has therefore been defined as '*the date on which maternity services had the first planned/structured contact with a pregnant woman to assess her history and needs so that local maternity services can provide further care such as an early pregnancy scan and antenatal screening tests*', i.e. the initial remote contact.

[1] https://tec.scot/clinical-specialty-guidance/

Available national data shows that, pre-COVID-19, at least 90% of pregnant woman attended their booking appointment by 12^{+6} weeks gestation[2]. There is currently no evidence that gestation at initial booking has increased due to COVID-19. If gestation and LMP are both missing on antenatal booking records, we will therefore assume the woman was at 12 weeks gestation at the date of booking.

For peer review only

[2] https://www.isdscotland.org/Health-Topics/Maternity-and-Births/Publications/

Defining start and end date of pregnancies

For pregnancies that have ended

Pregnancy end dates will be taken from end of pregnancy records as noted above

Pregnancy start date (date of conception) will be imputed from the pregnancy end date and the gestation at pregnancy end – 2 weeks

For continuing pregnancies

Pregnancy start date (date of conception) will be imputed from the date of antenatal booking and the gestation at booking – 2 weeks, or from the date of last menstrual period + 2 weeks if gestation is missing and LMP is provided

Time lags inherent in data sources identifying COVID-19 status and relevant outcomes

In general, the time lags inherent in data sources identifying COVID-19 status and relevant outcomes are less than (or at least no more than) those inherent in the various data sources required to identify pregnancy status.

The only additional lag that needs to be considered is that seen in Scottish Birth Record (SBR) records. SBR records are not returned to PHS as such. Rather, PHS takes a monthly download of data held on the system for analysis purposes. In most NHS Boards, the SBR system is used to generate a CHI number for a baby shortly after birth. Skeleton records with minimal demographic data are therefore available for all babies in a timely manner. For babies admitted to neonatal care, clinical coding staff within NHS Board admin departments are responsible for completing additional variables within a baby's SBR record following their discharge. There is no national standard for when this should be done and in practice the lag between discharge and a completed record being available varies between Boards. Some Boards achieve broadly complete records within 3 months whereas others take considerably longer. Currently (June 2020) NHS Borders and NHS Dumfries & Galloway have not coded any SBR records (or provided comparable data directly to PHS) since June 2017 and April 2018 respectively. SBR data is therefore unlikely to provide a complete picture of neonatal admissions within the timeframes set out for this analysis (i.e. for babies born in March 2020, the data available to PHS on SBR by July 2020 will only provide a partial picture of admissions to neonatal care). PHS may explore getting a new national feed from NHS Boards of more real time data on neonatal admissions to mitigate this problem if feasible.

For peer review only
